# Online Guide for Electronic Health Evaluation Approaches: Systematic Scoping Review and Concept Mapping Study

**DOI:** 10.2196/17774

**Published:** 2020-08-12

**Authors:** Tobias N Bonten, Anneloek Rauwerdink, Jeremy C Wyatt, Marise J Kasteleyn, Leonard Witkamp, Heleen Riper, Lisette JEWC van Gemert-Pijnen, Kathrin Cresswell, Aziz Sheikh, Marlies P Schijven, Niels H Chavannes, EHealth Evaluation Research Group

**Affiliations:** 1 Department of Public Health and Primary Care Leiden University Medical Centre Leiden Netherlands; 2 National eHealth Living Lab Leiden Netherlands; 3 Department of Surgery Amsterdam Gastroenterology and Metabolism Amsterdam UMC Amsterdam Netherlands; 4 Wessex Institute University of Southampton Southampton United Kingdom; 5 Department of Medical Informatics Amsterdam UMC Amsterdam Netherlands; 6 Ksyos Health Management Research Amstelveen Netherlands; 7 Department of Clinical, Neuro and Developmental Psychology Vrije Universiteit Amsterdam Netherlands; 8 Department of Psychology, Health and Technology Centre for eHealth and Wellbeing Research University of Twente Enschede Netherlands; 9 Centre of Medical Informatics Usher Institute The University of Edinburgh, Medical School Edinburgh United Kingdom; 10 Please see acknowledgements section for list of collaborators

**Keywords:** eHealth, mHealth, digital health, methodology, study design, health technology assessment, evaluation, scoping review, concept mapping

## Abstract

**Background:**

Despite the increase in use and high expectations of digital health solutions, scientific evidence about the effectiveness of electronic health (eHealth) and other aspects such as usability and accuracy is lagging behind. eHealth solutions are complex interventions, which require a wide array of evaluation approaches that are capable of answering the many different questions that arise during the consecutive study phases of eHealth development and implementation. However, evaluators seem to struggle in choosing suitable evaluation approaches in relation to a specific study phase.

**Objective:**

The objective of this project was to provide a structured overview of the existing eHealth evaluation approaches, with the aim of assisting eHealth evaluators in selecting a suitable approach for evaluating their eHealth solution at a specific evaluation study phase.

**Methods:**

Three consecutive steps were followed. Step 1 was a systematic scoping review, summarizing existing eHealth evaluation approaches. Step 2 was a concept mapping study asking eHealth researchers about approaches for evaluating eHealth. In step 3, the results of step 1 and 2 were used to develop an “eHealth evaluation cycle” and subsequently compose the online “eHealth methodology guide.”

**Results:**

The scoping review yielded 57 articles describing 50 unique evaluation approaches. The concept mapping study questioned 43 eHealth researchers, resulting in 48 unique approaches. After removing duplicates, 75 unique evaluation approaches remained. Thereafter, an “eHealth evaluation cycle” was developed, consisting of six evaluation study phases: conceptual and planning, design, development and usability, pilot (feasibility), effectiveness (impact), uptake (implementation), and all phases. Finally, the “eHealth methodology guide” was composed by assigning the 75 evaluation approaches to the specific study phases of the “eHealth evaluation cycle.”

**Conclusions:**

Seventy-five unique evaluation approaches were found in the literature and suggested by eHealth researchers, which served as content for the online “eHealth methodology guide.” By assisting evaluators in selecting a suitable evaluation approach in relation to a specific study phase of the “eHealth evaluation cycle,” the guide aims to enhance the quality, safety, and successful long-term implementation of novel eHealth solutions.

## Introduction

### Background

Electronic health (eHealth) solutions play an increasingly important role in the sustainability of future health care systems. An increase in the use and adoption of eHealth has been observed in the last decade. For instance, 59% of the member states of the European Union had a national eHealth record system in 2016 [1]. Despite the increase in use and high expectations about the impact of eHealth solutions, scientific evidence about the effectiveness, along with other aspects such as usability and accuracy, is often lagging behind [2-6]. In addition, due to rising demands such as time and cost restrictions from policymakers and commercial interests, the quality of eHealth evaluation studies is under pressure [7-9]. Although most eHealth researchers are aware of these limitations and threats, they may find it difficult to determine the most suitable evaluation approach to evaluate their novel eHealth solution since a clear overview of the wide array of evaluation approaches is lacking. However, to safely and successfully implement novel eHealth solutions into existing health care pathways, and to facilitate long-term implementation, robust scientific evaluation is paramount [10].

### Limitations of Classic Methodologies in eHealth Research

The most rigorous method to study the effects of health interventions is considered to be the double blinded parallel-group randomized controlled trial (RCT). Randomization has the unique ability to distribute both known and unknown confounders between study arms equally [11]. Although many RCTs of eHealth solutions have been published, limitations of this method are frequently described in the literature [12]. For instance, information bias could occur due to blinding difficulties because of the visibility of an eHealth solution [13-16]. Moreover, conducting an RCT can be very time-consuming, whereas eHealth technology develops rapidly. Consequently, before the trial results are known, the tested eHealth solution may be outdated [17]. Further, “contamination” in which the control group also uses a digital intervention, despite being randomized to the no-intervention group, easily occurs in eHealth research. Another drawback of placing too much focus on the classical research methodologies that are generally used to evaluate effectiveness is that the need for significant evaluation during the development and implementation phases of eHealth is often neglected. Additionally, validating the quality and evaluating behavioral aspects of an eHealth solution may be lacking [18,19]. Although it is not wrong to use classical research methods such as an RCT to study eHealth solutions, given the fact that eHealth solutions are considered to be “complex” interventions, more awareness about the wide array of eHealth evaluation approaches may be required.

### Evaluation of eHealth as a Complex Intervention

As described by the Medical Research Council (MRC) Framework 2000, eHealth solutions typically have multiple interacting components presenting several additional problems for evaluators, besides the already practical and methodological difficulties described above [20,21]. Because of these difficulties, eHealth solutions are considered as complex interventions. To study such interventions, multiple evaluation approaches are needed that are capable of answering the many different questions that arise during the consecutive phases of intervention development and implementation, including the “development,” “feasibility and piloting,” “evaluation,” and “implementation” phases [21]. For instance, to assess the effectiveness of complex interventions, the MRC Framework authors suggest the following experimental designs: individually randomized trials, cluster randomized trials, stepped wedge designs, preference trials, randomized consent designs, and N-of-1 designs. Unfortunately, the authors did not offer suggestions of evaluation approaches to use in the other phases of the MRC Framework. Murray et al [20] proposed a staged approach to the evaluation of eHealth that is modeled on the MRC Framework for Complex Interventions with 10 core questions to help developers quantify the costs, scalability, sustainability, and risks of harm of the eHealth solution. Greenhalgh et al [22] developed the Nonadoption, Abandonment, and challenges to Scale-up, Spread, and Sustainability (NASSS) framework to identify, understand, and address the interacting challenges around achieving sustained adoption, local scale-up, distant spread, and long-term sustainability of eHealth programs. Both of these studies illustrated and justified the necessity of a variety of evaluation approaches for eHealth beyond the RCT; however, this research does not assist evaluators in choosing which approach to use in a selected evaluation study phase. Another suggestion to improve the quality of eHealth research was proposed by Nykanen et al [23,24], who developed the guideline for Good Evaluation Practice in Health Informatics (GEP-HI), which precisely describes how to design and carry out a health informatics evaluation study in relation to the evaluation study phases. However, this guideline also did not include information on which specific evaluation approaches could be used in the related study phases. Besides the individual studies described above, there have been several books published concerning eHealth evaluation research. Among one of the first books on the topic is the “Handbook of Evaluation Methods for Health Informatics,” which was published in 2006 [25]. The aim of this book was to suggest options for finding appropriate tools to support the user in accomplishing an evaluation study. The book contains more than 30 evaluation methods, which are related to the phases of the system lifecycle, and the reliability, degree of difficulty, and resource requirements for each method are described. Moreover, the book “Evidence-Based Health Informatics,” published in 2016 [26], provides the reader with a better understanding of the need for robust evidence to improve the quality of health informatics. The book also provides a practical overview of methodological considerations for health information technology, such as using the best study design, stakeholder analysis, mixed methods, clinical simulation, and evaluation of implementation.

Although useful work has been performed by these previous authors, no single source is able to provide clear guidance in selecting appropriate evaluation approaches in relation to the specific evaluation phases of eHealth. Therefore, to enhance quality and safety, and to facilitate long-term implementation of eHealth solutions into daily practice, raising the awareness of eHealth evaluators about the wide array of eHealth evaluation approaches and thereby enhancing the completeness of evidence is sorely needed [27].

### Aim and Objectives

The overall aim of the present study was to raise awareness among eHealth evaluators about the wide array of eHealth evaluation approaches and the existence of multiple evaluation study phases. Therewith, quality, safety, and successful long-term implementation of novel eHealth solutions may be enhanced.

To achieve this aim, we pursued the following objectives: (1) systematically map the current literature and expert knowledge on methods, study designs, frameworks, and philosophical approaches available to evaluate eHealth solutions; and (2) provide eHealth evaluators with an online “eHealth methodology guide” to assist them with selecting a suitable evaluation approach to evaluate their eHealth solution in a specific study phase.

## Methods

### Overall Design

The project consisted of three consecutive steps: (1) a systematic scoping review, (2) concept mapping study, and (3) development of the "eHealth methodology guide" with content based on the results from steps 1 and 2.

### Step 1: Systematic Scoping Review

**Figure 1:**
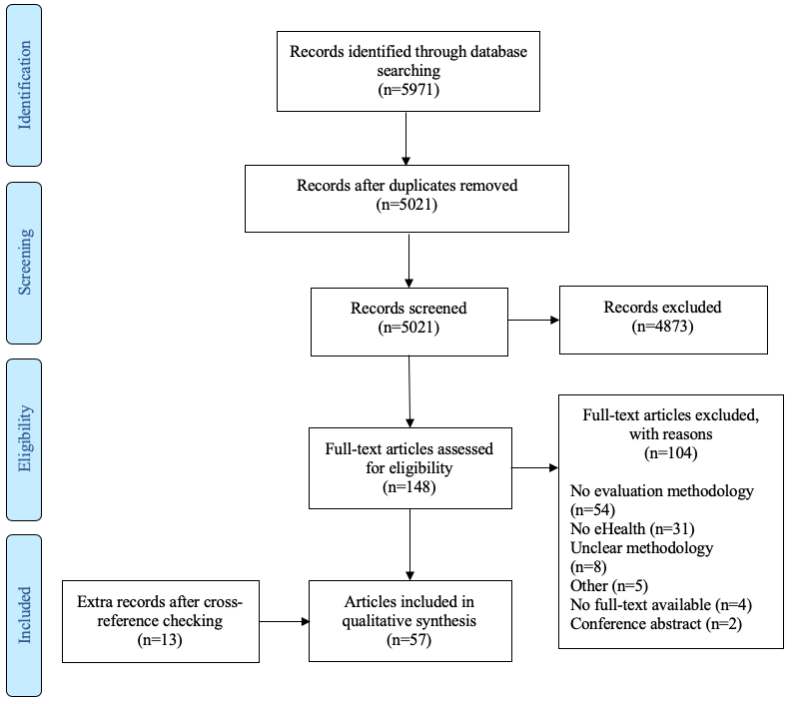
Preferred Reporting Items for Systematic Reviews and Meta-Analyses (PRISMA) flow diagram of the article selection process.

To describe the methods, study designs, frameworks, and other philosophical approaches (collectively referred to as “evaluation approach[es]”) currently used to evaluate eHealth solutions, a systematic scoping review was conducted. The online databases Pubmed, Embase, and PsycINFO were systematically searched using the term ”eHealth” in combination with ”evaluation” OR “methodology.” The search included Medical Subject Headings or Emtree terms and free-text terms. A complete list of the search strings is shown in Multimedia Appendix 1. Broad inclusion criteria were applied. All types of peer-reviewed English language articles published from January 1, 2006 until November 11, 2016 and a subsequent update from November 12, 2016 until October 21, 2018 describing any eHealth evaluation approach were included. We reasoned that articles published before January 1, 2006 would not necessarily need to be screened because the annual number of publications related to eHealth evaluation approaches was still low at that time, suggesting that the field was just starting to take its first scientific steps. In addition, if an article did describe a useful evaluation approach, it would have also been described by articles that were published later. Two reviewers (TB and AR) independently screened the titles and abstracts of the articles according to the inclusion criteria described above. Cohen kappa coefficient was calculated to measure the initial interrater reliability. Disagreements between the reviewers were resolved by the decision of a third independent reviewer (MK). Full-text assessment of the selected articles after screening of titles and abstracts was performed by both reviewers (TB and AR). Exclusion criteria after full-text assessment were: no eHealth evaluation approach described, article did not concern eHealth, the described methodology was unclear, full-text version was not available, or the article was a conference abstract. The reference list of eligible articles was checked for relevant additional studies. These studies were also checked for eligibility and included as crossreferenced articles in the Preferred Reporting Items for Systematic Reviews and Meta-analyses (PRISMA) diagram (Figure 1). In the qualitative synthesis, the eHealth evaluation approach was extracted from eligible articles, and duplicates and synonyms were merged to develop a single list of all the methods.

### Step 2: Concept Mapping Study

#### Overview of Phases

Although the systematic scoping review was performed rigorously, it was possible that not all of the current or possible approaches to evaluate eHealth solutions would have been described in the eligible studies. Therefore, to achieve a reasonably complete overview of eHealth evaluation approaches, it was considered essential to incorporate eHealth researchers’ knowledge on these approaches. A concept mapping study was selected as the most suitable method for structuring the suggested evaluation approaches from the researchers and for exploring their views on the different phases of the “eHealth evaluation cycle.” Concept mapping is a qualitative research methodology that was introduced by Trochim and Linton in 1986 [28]. It can be used by a group of individuals to first determine the scope of ideas on a certain topic and then to structure these ideas [29]. There is no interaction between the participants. A typical concept mapping study consists of 5 phases: (1) selection of the participants; (2) brainstorm, generation of the evaluation approaches by participants; (3) sorting and rating of the evaluation approaches; (4) concept mapping analysis; (5) and interpretation and utilization of the concept map. In the next subsections, these 5 phases are described in more detail. Concept System 2017 Global MAX online software was used for these tasks [30]. A Venn diagram was drawn to visualize the overlap between the results of the scoping review (step 1) and the evaluation approaches suggested by participants (step 2).

#### Selection of the Participants

To include a wide cross-section of eHealth researchers and reduce the influence of “group think,” any researchers in contact with the authors and with any level of expertise in eHealth or evaluation research (to help assure that all major perspectives on the eHealth evaluation topic were represented) were approached as being suitable participants for this concept mapping study. Snowball sampling (ie, asking participants to recruit other researchers) was also included in the recruitment strategy. The target participants received an email describing the objective of the study and instructions on how they could participate. A register was kept of the number of participants that were approached and that refused. In general, in a concept mapping study, there are no “rules” established as to how many participants should be included [31]. However, we estimated that 25 or more participants would be appropriate to generate a sufficient number of evaluation approaches and to have representative sorting and rating results.

#### Brainstorm Phase: Generation of the List of Evaluation Approaches

In this phase, participants were asked to enter all of the evaluation approaches they were aware of into an online form using Global MAX software. We intentionally did not include a strict definition of “evaluation approaches” so as to maintain the concept mapping phase as broad as possible and to avoid missing any methods due to an overly restrictive definition. The participants were not familiar with the results of the systematic scoping review. Participants were also asked 8 general background questions about their age, gender, background, years of experience in research, type of health care institute they work at, whether their daily work comprised eHealth, self-rated expertise in eHealth in general (grade 1-10), and self-rated expertise (grade 1-10) in eHealth evaluation approaches.

#### Sorting and Rating Phases

The coordinating researcher (AR) reviewed the evaluation approaches suggested by the participants, checking if each suggested approach truly represented a specific evaluation approach rather than, for instance, a broad methodological category such as “qualitative research.” If the coordinating researcher was unfamiliar with the suggested approach, Pubmed or Google Scholar was searched for supporting information. The cleaned results were combined with the results from the systematic scoping review, omitting duplicate approaches. The resulting set of approaches was then presented back to the participants who were instructed to sort these approaches into categories that had to be created by the participants. The participant was instructed to keep the following question in mind while sorting each approach into a self-created category: “To which phase of the research cycle (eg, planning, testing, implementation) does this evaluation approach belong?” To gain insights about opinions of the researchers with respect to the use in daily practice and suitability for effectiveness testing of the evaluation approaches, the participants were asked the following three rating questions about each approach: (1) Does your research group use this approach, or did it do so in the past? (yes or no); (2) In your opinion, how important is it that researchers with an interest in eHealth are familiar with this approach? (1, unimportant; 2, less important; 3, very important; 4, absolutely essential); (3) In your opinion, how important is the approach for proving the effectiveness of eHealth? (1, unimportant; 2, less important; 3, very important; 4, absolutely essential).

Results of the first rating question are reported as percentages of how many participants use or used the approach. For the second and third questions related to familiarity with the approach and importance for proving effectiveness, respectively, average rating scores ranging from 1 to 4 for each evaluation approach and the proportion of participants who selected categories 3 or 4 are reported.

#### Concept Mapping Analysis

Global MAX software uses a 3-step analysis to compute the concept map [32]. First, the sorting data from each participant were compiled into a similarity matrix. The matrix illustrates how many times each approach was sorted into similar categories. Second, the software applied a multidimensional scaling algorithm to plot points that were frequently sorted close together on a point map. A stress value (0-1), indicating the goodness of fit of the configuration of the point map, was calculated; the lower the stress value, the better the fit. In the last step, a hierarchical cluster analysis using the Ward algorithm was applied to group approaches into clusters (see also pages 87-100 of Kane and Trochim [33] for a detailed description of the data analyses to compute concept maps).

Two authors (TN and AR) reviewed the concept maps ranging from a 7-cluster to a 3-cluster option. The guidance of Kane and Trochim [33] was followed to select the best fitting number of clusters. Once the best fitting number of clusters was identified, each evaluation approach on the concept map was reviewed by the two authors to check if the approach truly belonged to the assigned cluster. If the approach seemed to belong in an adjacent cluster, it was reassigned to that particular cluster. If an approach could be assigned to multiple clusters, the best fitting cluster was selected.

The average rating scores for the rating questions on familiarity with the approach and importance for proving effectiveness were used to create a 4-quadrant Go-Zone graph. The Go-Zone graph easily visualizes the evaluation approaches with above-average rating scores on both questions, which are represented in the upper right quadrant. Approaches in the upper right quadrant that were also mentioned in the effectiveness testing cluster of the concept map are asterisked in the “eHealth methodology guide,” meaning that participants in general used these approaches and that these approaches were recommended by participants for evaluating effectiveness.

#### Interpretation and Utilization of the Concept Map

The initial concept map clusters represented names that participants suggested when sorting the evaluation approaches into self-created categories. Because these cluster names were used to constitute the phases of the “eHealth evaluation cycle” later in the project, three authors (TN, AR, and JW) determined (after multiple discussion sessions) the most appropriate names for the final concept map clusters. A name was found to be appropriate when it was suggested by multiple participants and was considered to be representative for the “eHealth evaluation cycle,” meaning that all of the evaluation approaches could be logically subdivided. After updating the names, the concept map clusters still contained the evaluation approaches allocated by the participants. This subdivision of eHealth evaluation approaches was used as the content for the “eHealth evaluation guide.”

### Step 3: eHealth Methodology Guide

The unique evaluation approaches identified in the systematic scoping review and unique evaluation approaches described by the participants in the concept mapping study were brought together by authors TB and AR, and used as the content to develop the “eHealth methodology guide.” To logically subdivide the eHealth evaluation approaches and to increase researchers’ awareness of the existence of multiple evaluation study phases, an “eHealth evaluation cycle” was developed. The cycle was based on the cluster names of the concept map and on the common denominators of the “all phases” evaluation approaches from the systematic scoping review. Each unique evaluation approach was assigned to a specific evaluation study phase. If an approach could belong to multiple study phases, it was assigned to all applicable phases.

## Results

### Step 1: Systematic Scoping Review

The systematic search retrieved 5971 articles from the databases. After removing duplicates, 5021 articles were screened using title and abstract review. A total of 148 articles were selected for full-text assessment. Among these, 104 articles were excluded because of the following reasons: not containing any named eHealth evaluation approach, not being an eHealth article, unclear description of approach, no full-text version available, conference abstract, and other reasons. Through crossreferencing, 13 additional articles were added to the final selection. In total, 57 articles were included in the qualitative synthesis. Calculation of Cohen kappa showed an interrater reliability of 0.49, which corresponds to “moderate agreement” between both reviewers. Figure 1 presents the PRISMA flow diagram describing the selection process. The 57 articles described 50 unique eHealth evaluation approaches (Table 1). Of the 50 methods, 19 were described by more than 1 article.

**Table 1:** Articles included in the systematic scoping review according to the evaluation approach adopted.

Reference	Year	Country	Evaluation approach
Chiasson et al [34]	2007	United Kingdom	Action research
Campbell and Yue [35]	2014	United States	Adaptive design; propensity score
Law and Wason [36]	2014	United Kingdom	Adaptive design
Mohr et al [16]	2015	United States	Behavioral intervention technology model (bit) in Trials of Intervention Principles; SMART^a^
Van Gemert-Pijnen et al [37]	2011	Netherlands	CeHRes^b^ Roadmap
Alpay et al [38]	2018	Netherlands	CeHRes Roadmap; Fog model; Oinas-Kukkonen model
Shaw [39]	2002	United Kingdom	CHEATS^c^: a generic ICT^d^ evaluation framework
Kushniruk and Patel [40]	2004	Canada	Cognitive task analysis; user-centered design
Jaspers [41]	2009	Netherlands	Cognitive walkthrough; heuristic evaluation; think-aloud method
Khajouei et al [42]	2017	Iran	Cognitive walkthrough; heuristic evaluation
Van Engen-Verheul et al [43]	2015	Netherlands	Concept mapping
Mohr et al [44]	2013	United States	CEEBIT^e^ framework
Nicholas et al [45]	2016	Australia	CEEBIT framework; single-case experiment (N=1)
Bongiovanni-Delaroziere and Le Goff Pronost [46]	2017	France	Economic evaluation; HAS^f^ methodological framework
Fatehi et al [47]	2017	Australia	Five-stage model for comprehensive research on telehealth
Baker et al [48]	2014	United States	Fractional-factorial (ANOVA^g^) design; SMART
Collins et al [49]	2007	United States	Fractional-factorial (ANOVA) design; MOST^h^; SMART
Chumbler et al [14]	2008	United States	Interrupted time-series analysis; matched cohort study design
Grigsby et al [50]	2006	United States	Interrupted time-series analysis; pretest-posttest design
Liu and Wyatt [51]	2001	United Kingdom	Interrupted time-series analysis
Kontopantelis et al [52]	2015	United Kingdom	Interrupted time-series analysis
Catwell and Shiekh [53]	2009	United Kingdom	Life cycle–based approach
Han [54]	2011	United States	Life cycle–based approach
Sieverink [55]	2017	Netherlands	Logfile analysis
Kramer-Jackman Popkess-Vawter [56]	2008	United States	Method for technology-delivered health care measures
Wilson et al [57]	2018	Canada	mHealth^i^ agile and user-centered research and development lifecycle
Jacobs and Graham[58]	2016	United States	mHealth development and evaluation framework; MOST
Dempsey et al [59]	2015	United States	Microrandomized trial; single-case experiment (N=1)
Klasnja et al [60]	2015	United States	Microrandomized trial; single-case experiment (N=1)
Law et al [61]	2016	United Kingdom	Microrandomized trial
Walton et al [62]	2018	United States	Microrandomized trial
Caffery et al [63]	2017	Australia	Mixed methods
Lee and Smith [64]	2012	United States	Mixed methods
Kidholm et al [65]	2017	Denmark	MAST^j^
Kidholm et al [66]	2018	Denmark	MAST
Kummervold et al [67]	2012	Norway	Noninferiority trial
May [68]	2006	United Kingdom	Normalization process theory and checklist
Borycki et al [69]	2016	Canada	Participatory design; user-centered design
Clemensen et al [70]	2017	Denmark	Participatory design
Glasgow [71]	2007	United States	Practical clinical trial; RE-AIM^k^ framework
Danaher and Seeley [72]	2007	United States	Pragmatic randomized controlled trial; SMART; Stage model of behavioral therapies research
Sadegh et al [73]	2018	Iran	Proposed framework for evaluated mHealth services
Harker and Kleinen [74]	2012	United Kingdom	Rapid review
Glasgow et al [75]	2014	United States	RE-AIM framework
Almirall et al [76]	2014	United States	SMART
Ammenwerth et al [77]	2012	Austria	Simulation study
Jensen et al [78]	2015	Denmark	Simulation study
Dallery et al [79]	2013	United States	Single case experiment (N=1)
Cresswell and Shiekh [80]	2014	United Kingdom	Sociotechnical evaluation
Kaufman et al [81]	2006	United States	Stead et al [82] evaluation framework
Brown and Lilford [83]	2006	United Kingdom	Stepped wedge (cluster) randomized trial
Hussey and Hughes [84]	2007	United States	Stepped wedge (cluster) randomized trial
Spiegelman [85]	2016	United States	Stepped wedge (cluster) randomized trial
Langbecker et al [86]	2017	Australia	Survey methods
Rönnby et al [87]	2018	Sweden	Technology acceptance model
Bastien [88]	2010	France	User-based evaluation
Nguyen et al [89]	2007	Canada	Waitlist control group design

^a^SMART: Sequential Multiple Assignment Randomized Trial.

^b^CeHRes: Centre for eHealth Research and Disease management.

^c^CHEATS: Clinical, human and organizational, educational, administrative, ethnical and social explanatory factors in a randomized controlled trial intervention.

^d^ICT: information and communication technology.

^e^CEEBIT: continuous evaluation of evolving behavioral intervention technology.

^f^HAS: Haute Autorité de Santé (French National Authority for Health).

^g^ANOVA: analysis of variance.

^h^MOST: multiphase optimization strategy.

^i^mHealth: mobile health.

^j^MAST: Model of Assessment of Telemedicine Applications.

^k^RE-AIM: Reach, Effectiveness, Adoption, Implementation, and Maintenance.

### Step 2: Concept Mapping Study

#### Characteristics of the Participants

In total, 52 researchers were approached to participate in the concept mapping study, 43 (83%) of whom participated in the “brainstorm” phase. Reasons for refusal to participate were a lack of time or not feeling skilled enough to contribute. From the 43 initial participants, 27 (63%) completed the “sorting” phase and 32 (74%) answered the three rating questions of the “rating” phase. The characteristics of participants for each phase are shown in Table 2. Participant characteristics did not change substantially throughout the study phases, with a mean participant age ranging from 39.9 to 40.5 years, a mean of 13 years of eHealth research experience, and more than 70% of participants working in a university medical center. The majority of participants gave themselves high grades for their knowledge about eHealth but lower scores for their expertise in eHealth evaluation approaches.

**Table 2:** Characteristics of study participants for each phase of the concept mapping study.

Characteristic		Brainstorm phase		Sorting phase		Rating phase	
Participants (n)		43^a^		27		32^b^	
Age (years), mean (SD)		39.9 (12.1)		39.0 (12.6)		40.5 (13)	
Female gender, n (%)		21 (49)		16 (53)		16 (50)	
Research experience (years), mean (SD)		13.5 (10.8)		12.6 (10.5)		13.9 (11)	
Working in university medical center, n (%)		37 (73)		26 (72)		27 (71)	
**Use of eHealth^c^** **in daily practice, n (%)**							
	During clinic work, not EHR^d^	4 (7)		3 (9)		3 (8)	
	During research	32 (59)		21 (60)		23 (59)	
	During clinic work and research	10 (19)		7 (20)		8 (21)	
	No	1 (2)		0 (0)		1 (3)	
	Other	7 (13)		4 (11)		4 (10)	
**Knowledge about eHealth, n (%)**							
	Grade 1-2	0 (0)		0 (0)		0 (0)	
	Grade 3-4	1 (2)		1 (4)		1 (3)	
	Grade 5-6	2 (5)		1 (4)		1 (3)	
	Grade 7-8	29 (71)		17 (63)		21 (68)	
	Grade 9-10	9 (22)		8 (30)		8 (26)	
**Expertise about eHealth research methods, n (%)**							
	Grade 1-2	0 (0)		0 (0)		0 (0)	
	Grade 3-4	1 (2)		1 (4)		1 (3)	
	Grade 5-6	15 (37)		8 (30)		11 (36)	
	Grade 7-8	19 (46)		15 (56)		15 (48)	
	Grade 9-10	6 (15)		3 (11)		4 (13)	
**Background, n (%)**							
	Biology	2 (3)		1 (2)		1 (2)	
	Data science	2 (3)		1 (2)		1 (2)	
	Economics	1 (1)		1 (2)		1 (2)	
	Medicine	24 (35)		14 (30)		18 (34)	
	(Health) Science	9 (13)		6 (13)		7 (13)	
	Industrial design	1 (1)		1 (2)		1 (2)	
	Informatics	4 (6)		3 (7)		3 (6)	
	Communication and culture	4 (6)		3 (7)		3 (6)	
	Psychology	14 (21)		11 (24)		12 (23)	
	Other	7 (10)		5 (11)		6 (11)	

^a^43 participants participated in the sorting phase, but 41 participants answered the characteristics questions.

^b^One of the 32 participants did not finish the third rating question: “importance for proving effectiveness.”

^c^eHealth: electronic health.

^d^EHR: electronic health record.

#### Brainstorm Phase

**Figure 2:**
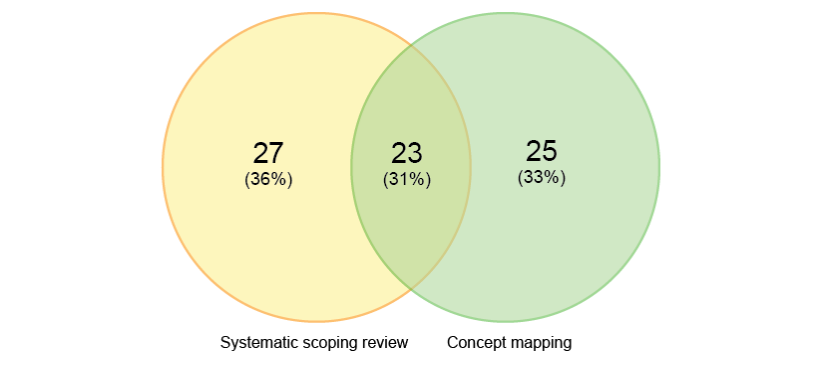
Venn diagram showing the origin of the 75 unique evaluation approaches.

Forty-three participants participated in an online brainstorm phase and generated a total of 192 evaluation approaches. After removing duplicate or undefined approaches, 48 unique approaches remained (Multimedia Appendix 2). Only 23 of these 48 approaches (48%) overlapped with those identified in the systematic scoping review (Figure 2).

Based on the update of the scoping literature review at the end of the project, 13 additional evaluation approaches were found that were not incorporated into the sorting and rating phases. Therefore, in total, only 62 of the 75 unique methods were presented to the participants in the sorting and rating phases. Participants were asked to sort the 62 evaluation approaches into as many self-created categories as they wished. Twenty-seven individuals participated in this sorting exercise, and they suggested between 4 and 16 categories each, with a mean of 8 (SD 4) categories.

**Figure 3:**
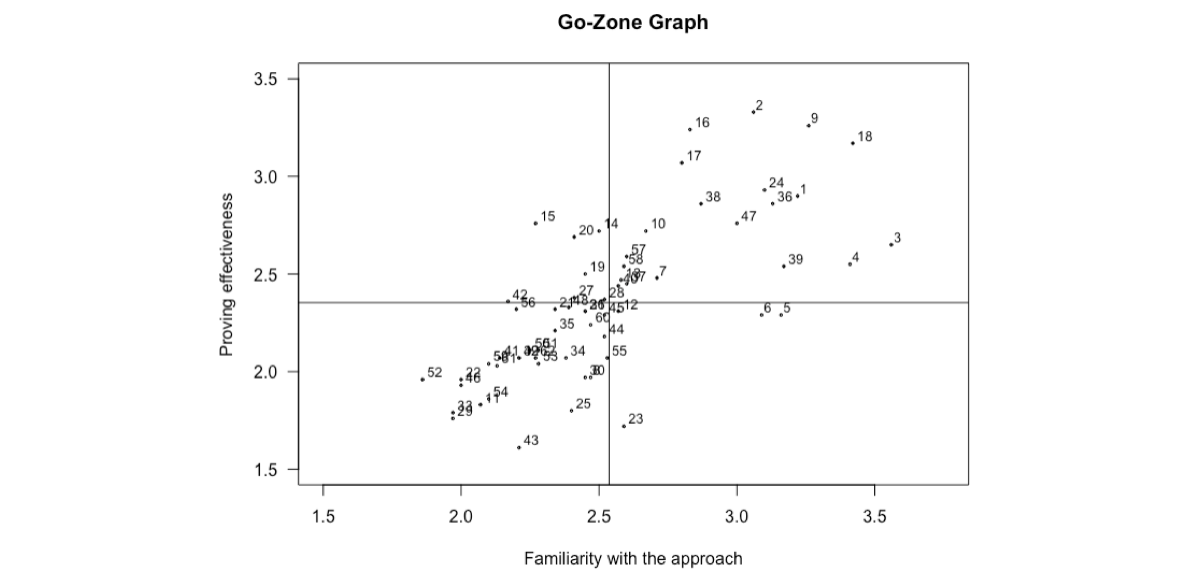
Go-Zone graph. The numbers refer to the evaluation approaches listed in Table 3.

The rating questions on use of the approach, familiarity with the approach, and importance for proving effectiveness were answered by 32, 32, and 31 participants, respectively. An analysis of responses to these three questions is presented in Table 3 and the mean ratings for familiarity with the approach and importance for proving effectiveness are plotted on the Go-Zone graph shown in Figure 3. The evaluation approach used most frequently by the participants was the questionnaire, with 100% responding “yes.” The approach that the participants used the least often was the Evaluative Questionnaire for E-health Tools at 3%. The average rating score for familiarity with the approach ranged from 1.9 for stage model of behavioral therapies to 3.6 for feasibility study. In addition, 88% of the participants thought that it is essential that researchers are familiar with the feasibility study method. The average rating score for importance for proving effectiveness ranged from 1.6 for vignette study to 3.3 for pragmatic RCT. In addition, 90% of the participants considered the stepped wedge trial design to be essential for proving the effectiveness of eHealth solutions.

**Table 3:** Results of step 2: concept mapping study.

Evaluation approach^a^		Use of approach^b^, % “yes” response	Familiarity with approach^c^		Proving effectiveness^d^	
			Mean	% of 3 + 4 (n/N)	Mean	% of 3 + 4 (n/N)
**Pilot/feasibility**		58 (SD 32.7)	2.9 (SD 0.5)		2.3 (SD 0.3)	
	3. Feasibility study^e^	94	3.6	88 (28/42)	2.6	52 (16/31)
	4. Questionnaire^e^	100	3.4	84 (27/63)	2.5	52 (16/31)
	8. Single-case experiments or n-of-1 study (N=1)	28	2.5	43 (13/60)	2.0	27 (8/30)
	12. Action research study	41	2.6	50 (15/58)	2.3	38 (11/29)
	44. A/B testing	25	2.5	45 (13/58)	2.2	36 (10/28)
**Development and usability**		37 (SD 29.1)	2.5 (SD 0.4)		2.1 (SD 0.3)	
	5. Focus group (interview)	91	3.2	81 (26/62)	2.3	32 (10/31)
	6. Interview	94	3.1	75 (24/62)	2.3	35 (11/31)
	23. Think-aloud method	66	2.6	52 (15/59)	1.7	14 (4/29)
	25. Cognitive walkthrough	31	2.4	37 (11/59)	1.8	17 (5/30)
	27. eHealth^f^ Analysis and Steering Instrument	12	2.4	55 (16/58)	2.4	48 (14/29)
	28. Model for Assessment of Telemedicine applications (MAST)	22	2.5	48 (14/59)	2.4	37 (11/30)
	29. Rapid review	31	2.0	23 (7/58)	1.8	7 (2/29)
	30. eHealth Needs Assessment Questionnaire (ENAQ)	6	2.4	45 (13/58)	2.0	24 (7/29)
	31. Evaluative Questionnaire for eHealth Tools (EQET)	3	2.4	52 (15/58)	2.3	41 (12/29)
	32. Heuristic evaluation	19	2.2	31 (9/57)	2.1	24 (7/29)
	33. Critical incident technique	9	2.0	24 (7/59)	1.8	4 (1/28)
	36. Systematic review^e^	94	3.1	67 (20/62)	2.9	69 (20/29)
	39. User-centered design methods^e^	53	3.2	73 (22/62)	2.5	50 (14/28)
	43. Vignette study	41	2.2	31 (9/58)	1.6	7 (2/28)
	45. Living lab	34	2.5	41 (12/58)	2.3	54 (15/28)
	50. Method for technology-delivered health care measures	9	2.3	39 (11/58)	2.1	25 (7/28)
	54. Cognitive task analysis (CTA)	16	2.1	23 (7/59)	1.9	18 (5/28)
	60. Simulation study	41	2.5	50 (15/60)	2.2	34 (10/29)
	62. Sociotechnical evaluation	22	2.3	37 (11/60)	2.1	29 (8/28)
**All phases**		11 (SD 4)	2.3 (SD 0.2)		2.2 (SD 0.2)	
	21. Multiphase Optimization Strategy (MOST)	6	2.3	45 (13/58)	2.3	39 (11/28)
	26. Continuous evaluation of evolving behavioral intervention technologies (CEEBIT) framework	6	2.4	48 (14/60)	2.3	38 (11/29)
	40. RE-AIM^g^ framework^e^	19	2.6	61 (17/59)	2.4	52 (14/27)
	46. Normalization process model	9	2.0	25 (7/57)	1.9	18 (5/28)
	48. CeHRes^h^ Roadmap	16	2.4	43 (12/58)	2.3	41 (11/27)
	49. Stead et al [82] evaluation framework	12	2.2	38 (11/58)	2.1	22 (6/27)
	51. CHEATS^i^: a generic information communication technology evaluation framework	6	2.3	41 (12/58)	2.1	26 (7/27)
	52. Stage Model of Behavioral Therapies Research	9	1.9	21 (6/58)	2.0	22 (6/27)
	53. Life cycle–based approach to evaluation	12	2.3	45 (13/58)	2.0	21 (6/28)
**Effectiveness testing**		45 (SD 23)	2.6 (SD 0.3)		2.6 (0.4)	
	1. Mixed methods^e^	87	3.2	81 (26/63)	2.9	65 (20/31)
	2. Pragmatic randomized controlled trial^e^	62	3.1	77 (24/63)	3.3	83 (25/30)
	7. Cohort study^e^ (retrospective and prospective)	81	2.7	58 (18/61)	2.5	58 (18/31)
	9. Randomized controlled trial^e^	91	3.3	71 (22/63)	3.3	74 (23/31)
	10. Crossover study^e^	44	2.7	57 (17/61)	2.7	59 (17/29)
	11. Case series	50	2.1	20 (6/60)	1.8	10 (3/29)
	13. Pretest-posttest study design^e^	62	2.6	45 (14/60)	2.5	50 (15/30)
	14. Interrupted time-series study	44	2.5	43 (13/59)	2.7	59 (17/29)
	15. Nested randomized controlled trial	31	2.3	37 (11/59)	2.8	55 (16/29)
	16. Stepped wedge trial design^e^	56	2.8	70 (21/60)	3.2	90 (26/29)
	17. Cluster randomized controlled trial^e^	50	2.8	60 (18/60)	3.1	69 (20/29)
	19. Trials of intervention principles (TIPs)^e^	23	2.5	42 (13/61)	2.5	43 (13/30)
	20. Sequential Multiple Assignment Randomized Trial (SMART)	9	2.4	45 (13/58)	2.7	62 (18/29)
	35. (Fractional-)factorial design	22	2.3	45 (13/58)	2.2	36 (10/28)
	37. Controlled before-after study (CBA)^e^	37	2.6	50 (15/60)	2.4	52 (15/29)
	38. Controlled clinical trial /nonrandomized controlled trial (CCT/NRCT)^e^	47	2.9	70 (21/60)	2.9	71 (20/28)
	41. Preference clinical trial (PCT)	19	2.1	24 (7/58)	2.1	25 (7/28)
	42. Microrandomized trial	9	2.2	24 (7/59)	2.4	50 (14/28)
	55. Cross-sectional study	72	2.5	40 (12/60)	2.1	29 (8/28)
	56. Matched cohort study	37	2.2	30 (9/59)	2.3	46 (13/28)
	57. Noninferiority trial design^e^	53	2.6	47 (14/60)	2.6	48 (14/29)
	58. Adaptive design^e^	19	2.6	52 (15/58)	2.5	50 (14/28)
	59. Waitlist control group design	34	2.1	28 (8/59)	2.0	32 (9/28)
	61. Propensity score methodology	31	2.1	30 (9/59)	2.0	21 (6/29)
**Implementation**		54 (SD 28)	2.8 (SD 0.5)		2.6 (SD 0.5)	
	18. Cost-effectiveness analysis	81	3.4	87 (27/63)	3.2	70 (21/30)
	22. Methods comparison study	16	2.0	17 (5/59)	2.0	21 (6/28)
	24. Patient reported outcome measures (PROMs)^e^	84	3.1	80 (24/60)	2.9	73 (22/30)
	34. Transaction logfile analysis	25	2.4	45 (13/57)	2.1	21 (6/28)
	47. Big data analysis^e^	62	3.0	73 (22/61)	2.8	59 (17/29)

^a^Approach identification numbers correspond with the numbers used in Figure 3 and Figure 4.

^b^Based on the rating question: “does your research group use this approach, or did it do so in the past?”; the percentage of “yes” responses is shown.

^c^Based on the rating question: “according to your opinion, how important is it that researchers with an interest in eHealth will become familiar with this approach?”; average rating scores ranging from unimportant (1) to absolutely essential (4) and percentages of categories 3 plus 4 are represented.

^d^The “proving effectiveness” column corresponds with the rating question: “according to your opinion, how important is the approach for proving the effectiveness of eHealth?” Average rating scores ranging from unimportant (1) to absolutely essential (4) and percentages of categories 3 plus 4 are presented.

^e^This approach scored above average on the rating questions “familiarity with the approach” and “proving effectiveness, ” which is plotted in the upper right quadrant of the Go-Zone graph (Figure 3).

^f^eHealth: electronic health.

^g^RE-AIM: Reach, Effectiveness, Adoption, Implementation, and Maintenance.

^h^CeHRes: Centre for eHealth Research and Disease management.

^i^CHEATS: Clinical, human and organizational, educational, administrative, ethnical and social explanatory factors in a randomized controlled trial intervention.

**Figure 4:**
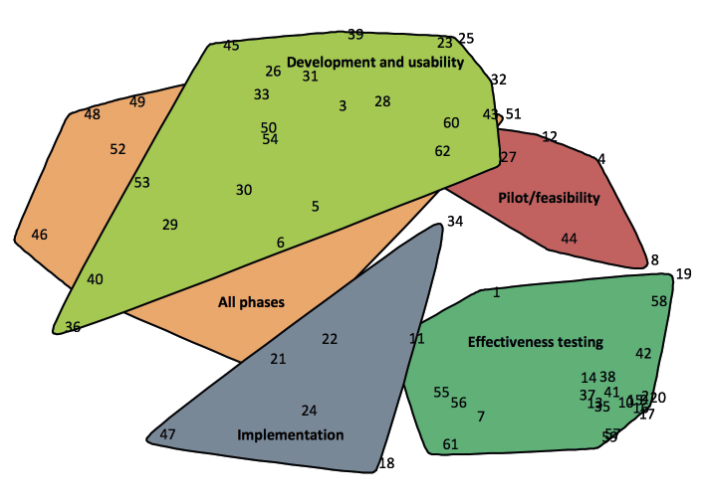
Concept map showing evaluation approaches grouped into five labeled clusters. The numbers refer to the approaches listed in Table 3.

#### Concept Mapping Analysis

Based on sorting data from 27 participants, a point map with a stress value of 0.27 was created. Compared with previous concept mapping study stress values, this represents a good fit [90,91]. In the next step, the software automatically clustered the points into the clusters shown on the concept map in Figure 4. A 5-cluster concept map was judged to represent the best fit for aggregating similar evaluation approaches into one cluster. Table 3 lists these clusters with average rating scores for the three rating questions and the approaches belonging in each cluster. With an average score of 2.9, the pilot/feasibility cluster showed the highest score on the familiarity with approach scale, whereas the “all phases” cluster showed the lowest average score at 2.3. With respect to responses to the importance for proving effectiveness question, the implementation cluster presented the highest average score at 2.6 and the development and usability cluster presented the lowest average score at 2.1.

Twenty of the 62 methods (32%) received above-average scores for both the questions related to familiarity with the approach and importance for proving effectiveness, and therefore appear in the upper right quadrant of the Go-Zone graph (Figure 3) and are indicated in Table 3. The majority of these approaches (12/20, 60%) fall into the effectiveness testing cluster.

#### Interpretation and Utilization of the Concept Mapping Study

The results of the concept map study were discussed within the team and the following names for the clusters were selected: “Development and usability,” “Pilot/feasibility,” “Effectiveness testing,” “Implementation,” and “All phases.”

### Step 3: eHealth Methodology Guide

Fifty evaluation approaches were identified in the systematic scoping review and 48 approaches were described by participants in the brainstorm phase of the concept mapping study. As visualized in the Venn diagram (Figure 2), 23 approaches were identified in both studies. Therefore, in total, 75 (50 + 48 – 23) unique evaluation approaches were identified. Examining the 23 approaches identified in both the literature and concept maps, 14 (67%) were described by more than one article.

**Figure 5:**
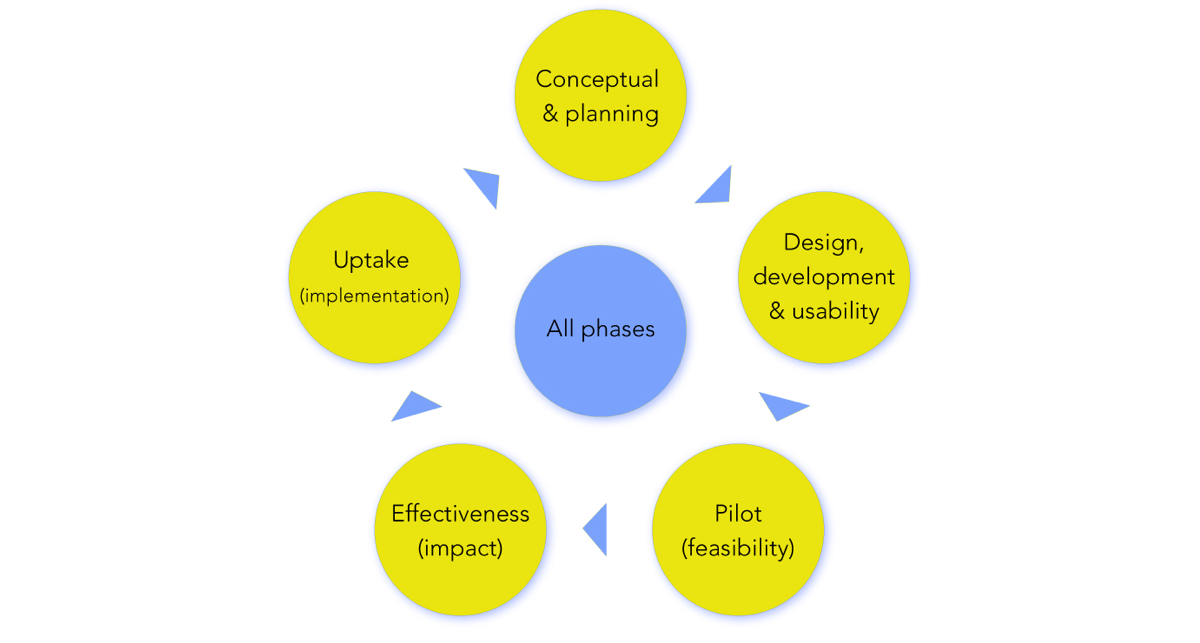
The “eHealth evaluation cycle” derived from empirical results of the scoping literature review and concept map study.

Based on the cluster names from the concept map (Figure 4), development and usability, pilot/feasibility, effectiveness testing, implementation, and the all phases evaluation approaches found in the systematic scoping review, an empirically based “eHealth evaluation cycle” was developed (Figure 5). The concept map did not reveal a conceptual and planning phase; however, based on the results of the systematic scoping review, and since there are evaluation approaches that belong to this phase, it was added to the “eHealth evaluation cycle.”

This evaluation cycle is iterative with consecutive evaluation study phases and an “all phases” cluster in the middle, which includes “all phases” evaluation frameworks such as Model for Assessment of Telemedecine that are capable of evaluating multiple study phases [65]. The “eHealth evaluation cycle” was used to construct the “eHealth methodology guide” by subdividing the guide into the evaluation study phase themes. Within the guide, each of the 75 unique evaluation approaches are briefly described and allocated to their respective evaluation study phase(s). Note that a single evaluation approach may belong to multiple evaluation phases.

The “eHealth methodology guide” can be found in Multimedia Appendix 3 and is available online [92]. Because the “eHealth methodology guide” is web-based, it is easy to maintain and, more importantly, it is easy to add content as new evaluation approaches may be proposed.

## Discussion

### Principal Findings

By carrying out a systematic scoping review and concept mapping study with eHealth researchers, we identified and aggregated 75 unique evaluation approaches into an online “eHealth methodology guide.” This online guide supports researchers in the field of eHealth to identify the appropriate study phase of the research cycle and choose an evaluation approach that is suitable for each particular study phase.

As stipulated by the participants in the concept mapping study, the most frequently used eHealth evaluation approaches were questionnaire (100%) and feasibility study (88%). The participants were most familiar with cost-effectiveness analysis (87%) and feasibility study (84%). In addition, they found pragmatic RCT (83%) and the stepped wedge trial design (90%) to be the most suitable approaches for proving effectiveness in eHealth research. Although a wide array of alternative evaluation approaches are already available, well-known traditional evaluation approaches, including all of the evaluation approaches described above, seemed to be most relevant for the participants. This suggests that eHealth research is still an immature field with too much focus on traditional evaluation approaches. However, to facilitate long-term implementation and safe use of novel eHealth solutions, evaluations performed by less-known evaluation approaches such as those described in the online “eHealth evaluation guide” are required.

The Go-Zone graph (Figure 3) confirms the practicing researchers’ familiarity with—and judged importance for proving the effectiveness of—the traditional evaluation approaches. The majority of the 20 approaches in the upper right quadrant of this graph are well-known study designs such as cohort study, (pragmatic) RCT, and controlled before-after study. Alternative and novel study designs (eg, instrumental variable analysis, interrupted time-series analysis) were not mentioned in the upper right quadrant, possible due to unfamiliarity.

### Comparison with Previous Work

Ekeland et al [93] performed a systematic review of reviews to summarize methodologies used in telemedicine research, analyze knowledge gaps, and suggest methodological recommendations for further research. They assessed and extracted data from 50 reviews and performed a qualitative summary and analysis of methodologies. They recommended that larger and more rigorous controlled studies are needed, including standardization of methodological aspects, to produce better evidence for the effectiveness of telemedicine. This is in line with our study, which provides easy access to, and an overview of, current approaches for eHealth evaluation throughout the research cycle. However, our work extends beyond effectiveness to cover the many other questions arising when developing and implementing eHealth tools. Aldossary et al [94] also performed a review to identify evaluations of deployed telemedicine services in hospitals, and to report methods used to evaluate service implementation. The authors included 164 papers describing 137 studies in the qualitative synthesis. They showed that 83 of the 137 studies used a descriptive evaluation methodology to report information about their activities, and 27 of the 137 studies evaluated clinical outcomes by the use of “traditional” study designs such as nonrandomized open intervention studies. Although the authors also reported methods to evaluate implementation, an overview of all evaluation study phases was lacking. In addition, no suggestions for alternative evaluation approaches were provided. Enam et al [27] developed an evaluation model consisting of multiple evaluation phases. The authors conducted a literature review to elucidate how the evidence of effectiveness and efficiency of eHealth can be generated through evaluation. They emphasized that generation of robust evidence of effectiveness and efficiency would be plausible when the evaluation is conducted through all distinct phases of eHealth intervention development (design, pretesting, pilot study, pragmatic trial, evaluation, and postintervention). This is partially in line with our study aim, and matches the “eHealth evaluation cycle” and online “eHealth methodology guide” developed as a result of our study. However, we added specific evaluation approaches to be used for each study phase and also incorporated other existing “all phases” research models.

### Strengths and Limitations

One of the greater strengths of this study was the combination of the scoping review and concept mapping study. The scoping review focused on finding eHealth-specific evaluation approaches. In contrast, in the concept mapping study, the participants were asked to write down any approach they were aware of that could contribute to the evaluation of eHealth. This slight discrepancy was intentional because we particularly wanted to find evaluation approaches that are actually being used in daily research practice to evaluate eHealth solutions. Therefore, the results from the systematic scoping review and the concept mapping study complement and reinforce each other, and therewith contribute to delivering a complete as possible “eHealth methodology guide.”

Another strength of this project was the level of knowledge and experience of the eHealth researchers who participated in the concept mapping study. They had approximately 13 years of eHealth research experience and the majority of participants graded themselves high for knowledge about eHealth. Interestingly, they gave themselves lower grades for their expertise in eHealth evaluation approaches. This means that we likely included an average group of eHealth researchers and did not only include the top researchers in the field of eHealth methodology. In our view, we had a representative sample of average eHealth researchers, who are also the target end users for our online “eHealth methodology guide.” This supports the generalizability and implementability of our project. However, the fact that more than 70% of participants worked in university medical centers may slightly limit the generalizability of our work to nonacademic researchers. It would be wise to keep an eye out for positive deviants outside university medical centers and users that are not senior academic “expert” eHealth researchers [95]. Slight wandering off the beaten track might be very necessary to find the needed innovative evaluation approaches and dissemination opportunities for sustainable implementation.

A limitation of our study was the date restriction of the systematic scoping review. We performed a broad systematic search but limited the search to only English language articles published from January 1, 2006 so as to keep the number of articles manageable. This could explain why some approaches, especially those published before 2006, were not found.

Another weakness of our study was that the systematic search was updated after the concept mapping exercise was complete. Therefore, 13 of the 75 evaluation approaches were not reviewed by the participants in the sorting and rating phases of the concept mapping study. However, this will also occur in the future with every new approach added to the online “eHealth methodology guide,” as the aim is to frequently update the guide.

### Future Perspectives

This first version of the “eHealth evaluation guide” contains short descriptions of the 75 evaluation approaches and references describing the approaches in more detail. Our aim is to include information on the level of complexity in the following version and other relevant resource requirements. Moreover, case example references will be added to the evaluation approaches to support the user in selecting an appropriate approach. Further, in the coming years, we aim to subject the “eHealth methodology guide” to an expert evaluation to assess the quality and ranking of the evaluation approaches, since this was not part of this present study. Finally, we are discussing collaboration and integration with the European Federation for Medical Informatics EVAL-Assessment of Health Information Systems working group.

### Conclusion

In this project, 75 unique eHealth evaluation approaches were identified in a scoping review and concept mapping study and served as content for the online “eHealth methodology guide.” The online “eHealth methodology guide” could be a step forward in supporting developers and evaluators in selecting a suitable evaluation approach in relation to the specific study phase of the “eHealth evaluation cycle.” Overall, the guide aims to enhance quality and safety, and to facilitate long-term implementation of novel eHealth solutions.

## Appendix

Multimedia Appendix 1 Search strategy.

Multimedia Appendix 2 List of 48 unique electronic health (eHealth) evaluation approaches suggested by participants of the concept mapping study.

Multimedia Appendix 3 eHealth methodology guide.

## Abbreviations

eHealth: electronic health
GEP-HI: Good Evaluation Practice in Health Informatics
MRC: Medical Research Council
NASS: Nonadoption, Abandonment, and challenges to Scale-up, Spread, and Sustainability
PRISMA: Preferred Reporting Items for Systematic Reviews and Meta-Analyses
RCT: randomized controlled trial
