# Correction: Online Guide for Electronic Health Evaluation Approaches: Systematic Scoping Review and Concept Mapping Study

**DOI:** 10.2196/23642

**Published:** 2020-08-21

**Authors:** Tobias N Bonten, Anneloek Rauwerdink, Jeremy C Wyatt, Marise J Kasteleyn, Leonard Witkamp, Heleen Riper, Lisette JEWC van Gemert-Pijnen, Kathrin Cresswell, Aziz Sheikh, Marlies P Schijven, Niels H Chavannes, EHealth Evaluation Research Group

**Affiliations:** 1 Department of Public Health and Primary Care Leiden University Medical Centre Leiden Netherlands; 2 National eHealth Living Lab Leiden Netherlands; 3 Department of Surgery Amsterdam Gastroenterology and Metabolism Amsterdam UMC Amsterdam Netherlands; 4 Wessex Institute University of Southampton Southampton United Kingdom; 5 Department of Medical Informatics Amsterdam UMC Amsterdam Netherlands; 6 Ksyos Health Management Research Amstelveen Netherlands; 7 Department of Clinical, Neuro and Developmental Psychology Vrije Universiteit Amsterdam Netherlands; 8 Department of Psychology, Health and Technology Centre for eHealth and Wellbeing Research University of Twente Enschede Netherlands; 9 Centre of Medical Informatics Usher Institute The University of Edinburgh, Medical School Edinburgh United Kingdom; 10 Please see acknowledgements section for list of collaborators

In “Online Guide for Electronic Health Evaluation Approaches: Systematic Scoping Review and Concept Mapping Study (“J Med Internet Res 2020;22(8):e17774”) the authors noted one error.

The metadata erroneously listed the group author "EHealth Evaluation Research Group" as having contributed equally. This has been corrected, and the group author is no longer listed as having contributed equally.

The correction will appear in the online version of the paper on the JMIR Publications website on August 21, 2020, together with the publication of this correction notice. Because this was made after submission to PubMed, PubMed Central, and other full-text repositories, the corrected article has also been resubmitted to those repositories

