# Supplementary material for: Online Guide for Electronic Health Evaluation Approaches: Systematic Scoping Review and Concept Mapping Study
Source: J Med Internet Res. 2020 Aug 12;22(8):e17774. doi: 10.2196/17774 (PMC7450369; doi:10.2196/17774)
Supplement: Multimedia Appendix 1 [file jmir_v22i8e17774_app1.docx]

# Multimedia Appendix 1

Search strategy

All searches were limited to English language and from date January 1, 2006, until October 21, 2018.

*Pubmed*: ("Telemedicine"[Mesh] OR "Medical Informatics"[Majr] OR "Internet"[Majr] OR telemedicine[tiab] OR ehealth[tiab] OR e-health[tiab] OR telehealth[tiab] OR medical informatics[tiab] OR mobile health[tiab] OR mhealth[tiab] OR m-Health[tiab]) AND ("Medicine"[Mesh] OR health) AND ("Research Design"[MAJR] OR "Randomised Controlled Trials as Topic/methods"[MAJR] OR "Evaluation Studies as Topic"[Mesh:NoExp] OR research method*[tiab] OR research strateg*[tiab] OR methodolog*[tiab]) AND (alternative*[tiab] OR effective*[tiab] OR evaluation*[tiab] OR quality[tiab]).

*Embase*: (exp *telemedicine/ or *medical informatics/ or *internet/ or (telemedicine or ehealth or e-health or telehealth or medical informatics or mobile health or mhealth or m-health).ti,ab.) and (exp medicine/ or health.ti,ab.) and (methodology/ or study design/ or *"randomised controlled trial (topic)"/ or "clinical trial (topic)"/ or evaluation study/ or (research method* or research strateg* or methodolog*).ti,ab.) and (alternative* or effective* or evaluation* or quality).ti,ab.

*PsycINFO*: (telemedicine/ or (telemedicine or ehealth or e-health or telehealth or medical informatics or mobile health or mhealth or m-health).ti,ab.) and (medical sciences/ or health.ti,ab.) and (experimental design/ or evaluation/ or program evaluation/ or (research method* or research strateg* or research design*.ti,ab. or methodolog*).ti,ab.) and (alternative* or effective* or evaluation* or quality).ti,ab
