# Supplementary material for: Online Guide for Electronic Health Evaluation Approaches: Systematic Scoping Review and Concept Mapping Study
Source: J Med Internet Res. 2020 Aug 12;22(8):e17774. doi: 10.2196/17774 (PMC7450369; doi:10.2196/17774)
Supplement: Multimedia Appendix 2 [file jmir_v22i8e17774_app2.docx]

# Multimedia Appendix 2

List of 48 unique eHealth evaluation approaches suggested by participants of the concept mapping study.

1 A/B testing

2 Action research

3 Big data analyze

4 Case series

5 Cluster randomized controlled trial

6 Cognitive walkthrough

7 Cohort study (retro- and prospective)

8 Continuous evaluation of evolving behavioral intervention technologies (CEEBIT) framework

9 Controlled before-and-after (CBA) study

10 Controlled clinical trial (CCT)

11 Cost-effectiveness analysis

12 Critical incident technique

13 Cross-sectional study

14 Crossover study

15 eHealth Analysis and Steeringinstrument (eASI)

16 eHealth Needs Assessment Questionnaire (ENAQ)

17 Evaluative Questionnaire for E-health Tools (EQET)

18 Feasibility study

19 Focus group

20 Fractional- factorial design

21 Heuristic evaluation

22 Interrupted time series analysis

23 Interview

24 Living lab

25 Logfile analysis

26 Methods comparison study

27 Micro-randomized trial

28 Mixed methods

29 Model for Assessment of Telemedicine applications (MAST)

30 Multiphase Optimization Strategy (MOST)

31 Normalization process model

32 Parallel cohort design with nested RCT

33 Patient reported outcome measures (PROMs)

34 Pragmatic randomized controlled trial

35 Preference clinical trial (PCT)

36 Pretest-posttest design

37 Questionnaire

38 Randomized controlled trial

39 Rapid review

40 RE-AIM framework

41 Sequential Multiple Assignment Randomized Trial (SMART)

42 Single-case experiments (N=1 trial)

43 Stepped wedge trial design

44 Systematic review

45 Think aloud method

46 Trials of intervention principles (TIPs)

47 User-centered design methods

48 Vignette study
