# Supplementary material for: Online Guide for Electronic Health Evaluation Approaches: Systematic Scoping Review and Concept Mapping Study
Source: J Med Internet Res. 2020 Aug 12;22(8):e17774. doi: 10.2196/17774 (PMC7450369; doi:10.2196/17774)
Supplement: Multimedia Appendix 3 [file jmir_v22i8e17774_app3.docx]

version: 1.0

eHealth methodology guide

Supplement belonging to original article:

*‘ An online guide for eHealth evaluation approaches: a systematic scoping review and concept mapping study’*

Doi: 10.2196/17774

Corresponding author:

Tobias N. Bonten
Leiden University Medical Centre
Department of Public Health & Primary Care, V6-22,
PO Box 9600, 2300 RC Leiden, the Netherlands.
Tel: +3171526; fax: +31715268444; Email: t.n.bonten@lumc.nl

# Background

The ‘eHealth methodology guide’ represents 75 unique evaluation methods, study designs, frameworks, and philosophical approaches (referred to as ‘evaluation approaches’) that are suitable to evaluate an eHealth solution in a certain evaluation study phase. Hereby the guide aims to support developers and researchers selecting an approach to evaluate their eHealth solution and to increased awareness of the existence of the multiple evaluation phases eHealth evaluation.

By performing a systematic scoping review and a concept mapping study with eHealth experts, 50 and 48 evaluation methods were identified respectively. Twenty-three methods were described by both studies. Therefore, in total, 50 + 48 – 23 = 75 unique evaluation approaches were identified and aggregated into this ‘eHealth methodology guide’, ordered by the ‘eHealth evaluation cycle’ as represented in the figure below.


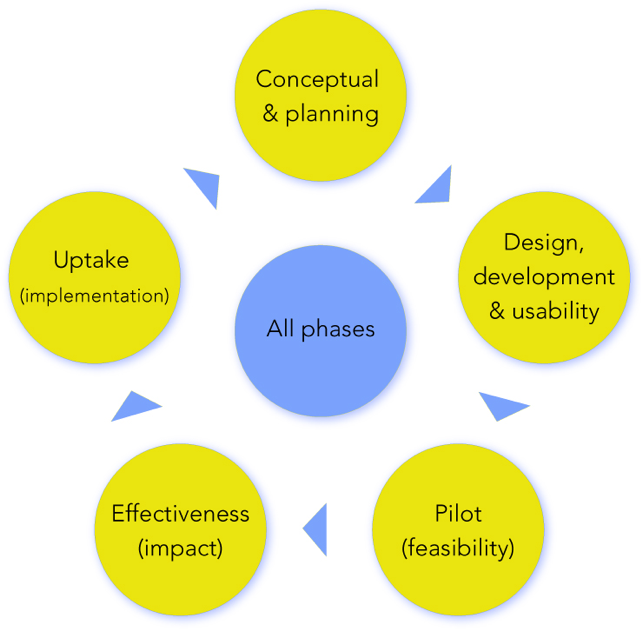


# How to use this guide?

The guide represents evaluation approaches in all the evaluation study phases. As an evaluator you should first select which evaluation phase you want to evaluate. Next, you go through the evaluation approaches of the selected phase and you decided upon the descriptions of the evaluation approaches and belonging references, which approach fits your eHealth solution best.

Approaches marked with an asterisk (*) scored above average in the rating exercise of the precedent concept mapping study. Meaning, that participants of the concept mapping study in general used these approaches more often and that these approaches are recommended by participants for evaluating effectiveness.

Table of content

Conceptual & planning 7

Concept mapping study^1^ 7

eHealth Needs Assessment Questionnaire (ENAQ)^2^ 7

Focus group^3,4^ 7

Interview^3^ 7

Living lab^5^ 7

Method for technology-delivered Healthcare Measures^6^ 7

Model of Oinas-Kukkonen^7^ 7

Survey methods^8^ 8

Rapid review^9^ 8

Systematic review^10^ 8

Think aloud method^11^ 8

Design, development & usability 9

A/B testing^12,13^ 9

Cognitive task analysis (CTA)^14^ 9

Cognitive walkthrough^11,15^ 9

Concept mapping study^1^ 9

Critical incident technique^16^ 9

eHealth Analysis and Steering Instrument (eASI)^17^ 9

eHealth Needs Assessment Questionnaire (ENAQ)^2^ 10

Focus group^3,4^ 10

Heuristic evaluation^11,15^ 10

Interview^3^ 10

Living lab^5^ 10

Method for technology-delivered Healthcare Measures^6^ 10

Mixed methods^32,33^ 10

Model of Fogg^7^ 11

Model of Oinas-Kukkonen^7^ 11

Participatory study^18,19^ 11

Survey methods^8^ 11

Simulation study^20,21^ 11

Systematic review^10^ 12

Think aloud method^11^ 12

Technology Acceptance Model (TAM)^22,23^ 12

User-based evaluation^24^ 12

User-centered design (UDC) methods^14,18^ 12

Vignette study^25^ 13

Pilot (feasibility) 14

A/B testing^12,13^ 14

Case series study^26^ 14

Cohort study (retro- and prospective) 14

Critical incident technique^16^ 14

Cross-sectional study^27^ 14

eHealth Analysis and Steering Instrument (eASI)^17^ 14

Evaluative Questionnaire for E-health Tools (EQET)^2^ 14

Feasibility study^28,29^ 15

Focus group^3,4^ 15

Interview^3^ 15

Living lab^5^ 15

Matched cohort study design^30^ 15

Method for technology-delivered Healthcare Measures^6^ 15

Methods comparison study^31^ 15

Mixed methods^32,33^ 16

Preference clinical trial (PCT)^34^ 16

Survey methods^8^ 16

Simulation study^20,21^ 16

Single-case experiment (N=1 trial)^35–38^ 17

Think aloud method^11^ 17

Vignette study^25^ 17

Effectiveness (impact) 18

Action research^39^ 18

Adaptive design^40,41^ 18

Big data analysis^42^ 18

Case series study^26^ 18

CHEATS: a generic information communication technology (ICT) evaluation^43^ 18

Cluster randomized controlled trial^44^ 18

Cohort study (retro- and prospective)^45^ 18

Controlled before-after study / non-randomized controlled trial (CBA / NRCT)^46^ 19

Controlled clinical trial (CCT)^47,48^ 19

Cost-effectiveness analysis^49,50^ 19

Cross-sectional study^27^ 19

Crossover study^51^ 19

Economic evaluation^52^ 19

(Fractional-)factorial (ANOVA) design^53,54^ 19

HAS methodological framework^52^ 19

Interrupted time series analysis^55–58^ 20

Matched cohort study design^30^ 20

Methods comparison study^31^ 20

Micro-randomised trial^36,3759,60^ 20

Mixed methods^32,33^ 20

Non-inferiority trial^61^ 21

Parallel cohort study with nested RCT^62^ 21

Patient reported outcome measures (PROMs)^63^ 21

Practical clinical trial (PCT)^64^ 21

Pragmatic randomised controlled trial (P-RCT)^65,66^ 21

Preference clinical trial (PCT)^34^ 22

Pretest-posttest study^56,67^ 22

Propensity score^40^ 22

Survey methods^8^ 22

Randomised controlled trial (RCT)^68^ 22

Sequential Multiple Assignment Randomised Trial (SMART)^53,54,65,69,70^ 23

Stepped wedge trial^71–73^ 23

Trials of intervention principles (TIPs)^70^ 23

Wait list control group study^74,75^ 23

Implementation (uptake) 24

Action research^39^ 24

Big data analysis^42^ 24

CHEATS: a generic information communication technology (ICT) evaluation^43^ 24

Economic evaluation^52^ 24

Critical incident technique^16^ 24

Focus group^3,4^ 24

HAS methodological framework^52^ 24

Interrupted time series analysis^55–58^ 25

Interview^3^ 25

Logfile analysis^76,77^ 25

Methods comparison study^31^ 25

Mixed methods^32,33^ 26

Normalization process model^78^ 26

Patient reported outcome measures (PROMs)^63^ 26

Survey methods^8^ 26

Sociotechnical evaluation^79^ 26

All phases methods 28

CeHRes Roadmap^80,81^ 28

Continuous evaluation of evolving behavioral intervention technologies (CEEBIT)^38,82^ 28

Five-stage model for comprehensive research on telehealth^83^ 28

Life-cycle–based approach^84^ 28

mHealth Agile and User-Centered Research and Development Lifecycle^85^ 28

mHealth Development and Evaluation Framework^86^ 28

Model for Assessment of Telemedicine applications (MAST)^87,88^ 29

Multiphase Optimization Strategy (MOST)^54,86^ 29

Proposed Framework for Evaluating mHealth Services^89^ 29

RE-AIM framework (Reach, Effectiveness, Adoption, Implementation and Maintenance)^64,90,91^ 29

Stage Model of Behavioral Therapies Research^65,92^ 29

Stead’s et al. evaluation framework^93,94^ 30

References 31

Appendix 38

# Conceptual & planning

## Concept mapping study[1]

Concept mapping methodology overcomes the drawbacks of qualitative study designs by integrating results from qualitative group sessions with multivariate statistical analysis to represent ideas of diverse stakeholders visually on maps. As the method is purposefully designed to integrate input from larger groups of participants with differing content expertise or interest in a domain in an efficient way and short time frame.

## eHealth Needs Assessment Questionnaire (ENAQ)[2]

The E-health Needs Assessment Questionnaire (ENAQ) is useful to map the general needs of older adults with low health literacy regarding eHealth.

## Focus group[3,4]

A focus group is a group discussion on a particular topic organized for research purposes. This discussion is guided, monitored and recorded by a researcher (sometimes called a moderator or facilitator). Focus groups are used for generating information on collective views, and the meanings that lie behind those views.

## Interview[3]

There are three fundamental types of research interviews: structured, semi-structured and unstructured. Structured interviews are, essentially, verbally administered questionnaires, in which a list of predetermined questions are asked, with little or no variation and with no scope for follow-up questions to responses that warrant further elaboration. Conversely, unstructured interviews do not reflect any preconceived theories or ideas and are performed with little or no organization. Semi-structured interviews consist of several key questions that help to define the areas to be explored, but also allows the interviewer or interviewee to diverge in order to pursue an idea or response in more detail.

## Living lab[5]

A Living Lab is a user-centered, open innovation ecosystem based on a systematic user co-creation approach, integrating research and innovation processes in real-life communities and settings.

## Method for technology-delivered Healthcare Measures[6]

The Method for Technology-delivered Healthcare Measures is designed to systematically guide the development and evaluation of technology-delivered measures. The five-step Method for Technology-delivered Healthcare Measures includes establishment of content, e-Health literacy, technology delivery, expert usability, and participant usability.

## Model of Oinas-Kukkonen[7]

The model of Oinas-Kukkonen includes principles for persuasive design and describes the key issues behind them. The model allows defining the persuasive context, describing the targeted users, their goals, intentions and technology use.

## Survey methods[8]*

Surveys are commonly used in telehealth research to assess patient satisfaction, patient experiences, patient preferences and attitudes, and the technical quality of a teleconsultation. The popularity of the survey as a method of measurement can be understood through three major strengths of this technique. First, confidential survey questions are well suited to capture individuals’ experiences, perceptions and attitudes. Second, pre-existing scales can be used across studies, enabling the comparison and replication of results. Third, the validity and reliability of survey instruments can be assessed through rigorous, transparent and well-accepted validation methods, providing the researcher with confidence that the measures tap the intended constructs, and provide an accurate measurement.

## Rapid review[9]

The term ‘RR’ does not appear to have one single definition but is framed in the literature as utilizing various stipulated time frames between 1 and 6 months. The word ‘rapid’ indicates that it will be carried out quickly, although this labelling does not inform us as to exactly which part of the review is intended to be carried out at a faster pace than a full SR. The name could imply the manipulation of agreed SR processes such as quicker searching and searching fewer databases, faster inclusion screening and/or having a narrower remit for inclusion of studies, limiting data extraction or analyzing the data by using only selected methods of quantitative or qualitative analysis in order to draw rapid conclusions about a specific research question. Indeed, it seems that any or all of these specifications could be applied to a RR in order to draw fast conclusions about a specific health intervention.

## Systematic review[10]*

A systematic review summarizes the results of available carefully designed healthcare studies (controlled trials) and provides a high level of evidence on the effectiveness of healthcare interventions. Judgments may be made about the evidence and inform recommendations for healthcare.

These reviews are complicated and depend largely on what clinical trials are available, how they were carried out (the quality of the trials) and the health outcomes that were measured. Review authors pool numerical data about effects of the treatment through a process called meta-analyses. Then authors assess the evidence for any benefits or harms from those treatments. In this way, systematic reviews are able to summarize the existing clinical research on a topic.

## Think aloud method[11]

The think aloud method can be of high value in evaluating a system's design on usability flaws and is therefore frequently used to gather information about a system's usability in testing computer systems with potential end users. During recorded usability sessions, users ‘interact’ with a (prototype) system or interface according to a predetermined set of scenarios while verbalizing their thoughts. Analyses of these verbal reports provide detailed insight into usability problems actually encountered by end users but also in the causes underlying these problems.

# Design, development & usability

## A/B testing[12,13]

A/B testing (also known as split testing or bucket testing) is a method of comparing two versions of a webpage or app against each other to determine which one performs better. AB testing is essentially an experiment where two or more variants of a page are shown to users at random, and statistical analysis is used to determine which variation performs better for a given conversion goal.

## Cognitive task analysis (CTA)[14]

CTA has been applied in the design of systems in order to create a better understanding of human information needs in development of systems. It categorizes tasks and observes patients (or other test persons) while performing these tasks (e.g. usage of an eHealth application).

## Cognitive walkthrough[11,15]

The cognitive walkthrough method is a type of usability evaluation technique that focuses on evaluating an (early) system design for learnability by exploration. In a cognitive walkthrough, an evaluator, preferably a usability expert evaluates a user interface by analyzing the cognitive processes required for accomplishing tasks that users would typically carry out supported by the computer.

## Concept mapping study[1]

Concept mapping methodology overcomes the drawbacks of qualitative study designs by integrating results from qualitative group sessions with multivariate statistical analysis to represent ideas of diverse stakeholders visually on maps. As the method is purposefully designed to integrate input from larger groups of participants with differing content expertise or interest in a domain in an efficient way and short time frame.

## Critical incident technique[16]

First described by John C. Flanagan in 1954, the critical incident technique (CIT) is a well-established qualitative research tool used in many areas of the health sciences. Flanagan describes the technique as consisting of “a set of procedures for collecting direct observations of human behavior in such a way as to facilitate their potential usefulness in solving practical problems.” The CIT began its life as an offshoot of the Aviation Psychology Program of the United States Army Air Forces in World War II.

## eHealth Analysis and Steering Instrument (eASI)[17]

The eASI surveys how eHealth services score on 3 dimensions (i.e., utility, usability, and content) and 12 underlying categories (i.e., insight in health condition, self-management decision making, performance of self-management, involving the social environment, interaction, personalization, persuasion, description of health issue, factors of influence, goal of eHealth service, implementation, and evidence).

## eHealth Needs Assessment Questionnaire (ENAQ)[2]

The E-health Needs Assessment Questionnaire (ENAQ) is useful to map the general needs of older adults with low health literacy regarding e-health.

## Focus group[3,4]

A focus group is a group discussion on a particular topic organized for research purposes. This discussion is guided, monitored and recorded by a researcher (sometimes called a moderator or facilitator). Focus groups are used for generating information on collective views, and the meanings that lie behind those views.

## Heuristic evaluation[11,15]

Among the usability inspection methods, heuristic evaluation is the most common and most popular. In a heuristic evaluation, a small set of evaluators inspects a system and evaluates its interface against a list of recognized usability principles—the heuristics. Typically, these heuristics are general principles, which refer to common properties of usable systems. Heuristic evaluation is in its most common form based on the following set of usability principles: (1) use simple and natural dialogue, (2) speak the user's language, (3) minimize memory load, (4) be consistent, (5) provide feedback, (6) provide clearly marked exits, (7) provide shortcuts, (8) provide good error messages, (9) prevent errors, and (10) provide help and documentation.

## Interview[3]

There are three fundamental types of research interviews: structured, semi-structured and unstructured. Structured interviews are, essentially, verbally administered questionnaires, in which a list of predetermined questions are asked, with little or no variation and with no scope for follow-up questions to responses that warrant further elaboration. Conversely, unstructured interviews do not reflect any preconceived theories or ideas and are performed with little or no organization. Semi-structured interviews consist of several key questions that help to define the areas to be explored, but also allows the interviewer or interviewee to diverge in order to pursue an idea or response in more detail.

## Living lab[5]

A Living Lab is a user-centered, open innovation ecosystem based on a systematic user co-creation approach, integrating research and innovation processes in real-life communities and settings.

## Method for technology-delivered Healthcare Measures[6]

The Method for Technology-delivered Healthcare Measures is designed to systematically guide the development and evaluation of technology-delivered measures. The five-step Method for Technology-delivered Healthcare Measures includes establishment of content, e-Health literacy, technology delivery, expert usability, and participant usability.

## Mixed methods[18,19]*

Mixed methods research (MMR) is an emerging and evolving research methodology that requires both qualitative and quantitative approaches within the same study. It is an approach to research in the social, behavioral and health sciences in which the investigator gathers both quantitative and qualitative data, integrates the two, and then draws interpretations based on the combined strengths of both sets of data to understand research problems. MMR is important for telehealth research because questions that profit most from a mixed methods design tend to be broad, complex and multifaceted.

## Model of Fogg[7]

The model is useful for understanding human behavior and to operationalize the factors related to it. It is applicable when designing persuasive technologies. The model of Fogg is relevant when developing eHealth self-management systems since behavioral changes reside at the core of such systems.

## Model of Oinas-Kukkonen[7]

The model of Oinas-Kukkonen includes principles for persuasive design and describes the key issues behind them. The model allows defining the persuasive context, describing the targeted users, their goals, intentions and technology use.

## Participatory study[20,21]

Participatory Design (PD) is one way of involving users and other stakeholders during the design phase. Three issues dominate PD: 1) the philosophy and politics behind the design concept, 2) the tools and techniques, and 3) the ability of the approach to provide a realm for understanding the socio-technical context and business strategic aims where the design solutions are to be applied. A core principle of PD is that users and other stakeholders are actively participating in design activities, where they have the power to influence the design solutions, and that they participate on equal terms.

## Survey methods[8]*

Surveys are commonly used in telehealth research to assess patient satisfaction, patient experiences, patient preferences and attitudes, and the technical quality of a teleconsultation. The popularity of the survey as a method of measurement can be understood through three major strengths of this technique. First, confidential survey questions are well suited to capture individuals’ experiences, perceptions and attitudes. Second, pre-existing scales can be used across studies, enabling the comparison and replication of results. Third, the validity and reliability of survey instruments can be assessed through rigorous, transparent and well-accepted validation methods, providing the researcher with confidence that the measures tap the intended constructs, and provide an accurate measurement.

## Simulation study[22,23]

A simulation or a simulator may be defined as a device ‘that attempts to re-create characteristics of the real world’. Study results show that full scale simulation studies are a useful method for testing the feasibility of information systems especially when taking into account the resources spent. Clinical simulation covers only part of the range of tests which should be conducted, and it should not be a substitute for a pilot implementation test in real settings. However, it is possible to use clinical simulations to gain important knowledge concerning work practices, usability and human factors prior to widespread system release, and they can thereby contribute greatly to ensuring patient safety.

## Systematic review[10]*

A systematic review summarizes the results of available carefully designed healthcare studies (controlled trials) and provides a high level of evidence on the effectiveness of healthcare interventions. Judgments may be made about the evidence and inform recommendations for healthcare.

These reviews are complicated and depend largely on what clinical trials are available, how they were carried out (the quality of the trials) and the health outcomes that were measured. Review authors pool numerical data about effects of the treatment through a process called meta-analyses. Then authors assess the evidence for any benefits or harms from those treatments. In this way, systematic reviews are able to summarize the existing clinical research on a topic.

## Think aloud method[11]

The think aloud method can be of high value in evaluating a system's design on usability flaws and is therefore frequently used to gather information about a system's usability in testing computer systems with potential end users. During recorded usability sessions, users ‘interact’ with a (prototype) system or interface according to a predetermined set of scenarios while verbalizing their thoughts. Analyses of these verbal reports provide detailed insight into usability problems actually encountered by end users but also in the causes underlying these problems.

## Technology Acceptance Model (TAM)[24,25]

The TAM is an information technology framework for understanding users’ adoption and use of emerging technologies particularly in the workplace environment. The theory posits that a person’s intent to use (acceptance of technology) and usage behavior (actual use) of a technology is predicated by the person’s perceptions of the specific technology’s usefulness (benefit from using the technology) and ease of use.

## User-based evaluation[26]

User-based evaluations are usability evaluation methods in which users directly participate. Users are invited to do typical tasks with a product, or simply asked to explore it freely, while their behaviors are observed and recorded in order to identify design flaws that cause user errors or difficulties.

## User-centered design (UDC) methods[14,20]

User-centered design is an approach to the design of information systems characterized as follows: (1) an early and continual focus on end users, (2) the empirical evaluation of systems, and (3) application of iterative design processes. As part of user-centered design, usability testing of systems has become a key method for carrying out empirical evaluation of designs from the end user’s perspective. Results from iterative and continued usability testing of early system designs, prototypes, and near completed systems can reveal a range of usability problems and areas where systems can be optimized and improved during the design process and before finalization of the system.

## Vignette study[27]

A quantitative vignette study consists of two components: (a) a vignette experiment as the core element, and (b) a traditional survey for the parallel and supplementary measurement of additional respondent-specific characteristics, which are used as covariates in the analysis of vignette data. A vignette is a short, carefully constructed description of a person, object, or situation, representing a systematic combination of characteristics. Within vignette studies, respondents are typically confronted not only with one single vignette but with a whole population of vignettes in order to elicit their beliefs, attitudes, judgments, knowledge, or intended behavior with respect to the presented vignette scenarios. Finally, the aim of a vignette study is to identify and assess the importance of those vignette factors which causally affect individual responses to the contextualized but hypothetical vignette settings.

# Pilot (feasibility)

## A/B testing[12,13]

A/B testing (also known as split testing or bucket testing) is a method of comparing two versions of a webpage or app against each other to determine which one performs better. AB testing is essentially an experiment where two or more variants of a page are shown to users at random, and statistical analysis is used to determine which variation performs better for a given conversion goal.

## Case series study[28]

Observational study design which describes several patients (cases) over time. Mostly hypothesis forming (early stage of effectiveness research) and without control group or placebo.

## Cohort study (retro- and prospective)[45]*

Observational design, in which groups of patients are followed over time. Usually, multiple exposures and outcomes can be defined in a cohort. Retro-and prospective mostly refers to the timing of data acquisition (before or after designing the study). Patients are sampled on the basis of exposure. Information about baseline characteristics is obtained, and the occurrence of outcomes is assessed during a specified follow-up period. At baseline, all exposed or unexposed persons or both may be included.

## Critical incident technique[16]

First described by John C. Flanagan in 1954, the critical incident technique (CIT) is a well-established qualitative research tool used in many areas of the health sciences. Flanagan describes the technique as consisting of “a set of procedures for collecting direct observations of human behavior in such a way as to facilitate their potential usefulness in solving practical problems.” The CIT began its life as an offshoot of the Aviation Psychology Program of the United States Army Air Forces in World War II.

## Cross-sectional study[29]

Observational study design, which samples the exposure and outcome at one moment in time. Useful to get quick insight in possible associations. Drawback is the lack of follow-up time to study relations between exposure and outcome over time.

## eHealth Analysis and Steering Instrument (eASI)[17]

The eASI surveys how eHealth services score on 3 dimensions (i.e., utility, usability, and content) and 12 underlying categories (i.e., insight in health condition, self-management decision making, performance of self-management, involving the social environment, interaction, personalization, persuasion, description of health issue, factors of influence, goal of eHealth service, implementation, and evidence).

## Evaluative Questionnaire for E-health Tools (EQET)[2]

The Evaluative Questionnaire for E-health Tools (EQET) can be used to assess the suitability of e-health applications for older adults with low health literacy.

## Feasibility study[30,31]*

Feasibility Studies are pieces of research done before a main study. They are used to estimate important parameters that are needed to design the main study. For instance: standard deviation of the outcome measure, which is needed in some cases to estimate sample size; willingness of participants to be randomized, willingness of clinicians to recruit participants, number of eligible patients. Crucially, feasibility studies do not evaluate the outcome of interest; that is left to the main study.

## Focus group[3,4]

A focus group is a group discussion on a particular topic organized for research purposes. This discussion is guided, monitored and recorded by a researcher (sometimes called a moderator or facilitator). Focus groups are used for generating information on collective views, and the meanings that lie behind those views.

## Interview[3]

There are three fundamental types of research interviews: structured, semi-structured and unstructured. Structured interviews are, essentially, verbally administered questionnaires, in which a list of predetermined questions are asked, with little or no variation and with no scope for follow-up questions to responses that warrant further elaboration. Conversely, unstructured interviews do not reflect any preconceived theories or ideas and are performed with little or no organization. Semi-structured interviews consist of several key questions that help to define the areas to be explored, but also allows the interviewer or interviewee to diverge in order to pursue an idea or response in more detail.

## Living lab[5]

A Living Lab is a user-centered, open innovation ecosystem based on a systematic user co-creation approach, integrating research and innovation processes in real-life communities and settings.

## Matched cohort study design[32]

Matching is not uncommon in epidemiological studies and refers to the selection of unexposed subjects’ i.e., controls that in certain important characteristics are identical to cases. Most frequently matching is used in case-control studies but it can also be used in cohort studies. The matching procedure is often directed towards classical background factors such as sex and age.

## Method for technology-delivered Healthcare Measures[6]

The Method for Technology-delivered Healthcare Measures is designed to systematically guide the development and evaluation of technology-delivered measures. The five-step Method for Technology-delivered Healthcare Measures includes establishment of content, e-Health literacy, technology delivery, expert usability, and participant usability.

## Methods comparison study[33]

Two different overarching methodologies for method-comparison studies have been commonly used: equivalence studies and non-inferiority studies. In equivalence studies, we are interested in whether the new assessment does not differ from the conventional (usually in-person) assessment in either direction by a pre-specified amount (i.e. a two-sided test). In an equivalence trial the new assessment method will be selected regardless of whether it is better or worse than an existing assessment as long as the difference falls within the predefined zone of allowable difference (and meets other criteria such as cost effective and stakeholder satisfaction). Commonly in telehealth, the existing model of care (e.g. specialist assessment in tertiary hospital for cognitive impairment) will not be replaced, but rather the telehealth option will be used for people who cannot access conventional services. In this case, the question is whether the telehealth assessment is ‘as good’ as or rather ‘not inferior’ to conventional practice.

## Mixed methods[18,19]*

Mixed methods research (MMR) is an emerging and evolving research methodology that requires both qualitative and quantitative approaches within the same study. It is an approach to research in the social, behavioral and health sciences in which the investigator gathers both quantitative and qualitative data, integrates the two, and then draws interpretations based on the combined strengths of both sets of data to understand research problems. MMR is important for telehealth research because questions that profit most from a mixed methods design tend to be broad, complex and multifaceted.

## Preference clinical trial (PCT)[34]

In a preference clinical trial (PCT), two or more health-care interventions are compared among several groups of patients, at least some of whom have purposefully chosen the intervention to be administered to them. This stands in contrast to the randomized, controlled clinical trial (RCT), where patients are randomly assigned to receive one of the available test interventions.

## Survey methods[8]*

Surveys are commonly used in telehealth research to assess patient satisfaction, patient experiences, patient preferences and attitudes, and the technical quality of a teleconsultation. The popularity of the survey as a method of measurement can be understood through three major strengths of this technique. First, confidential survey questions are well suited to capture individuals’ experiences, perceptions and attitudes. Second, pre-existing scales can be used across studies, enabling the comparison and replication of results. Third, the validity and reliability of survey instruments can be assessed through rigorous, transparent and well-accepted validation methods, providing the researcher with confidence that the measures tap the intended constructs, and provide an accurate measurement.

## Simulation study[22,23]

A simulation or a simulator may be defined as a device ‘that attempts to re-create characteristics of the real world’. Study results show that full scale simulation studies are a useful method for testing the feasibility of information systems especially when taking into account the resources spent. Clinical simulation covers only part of the range of tests which should be conducted, and it should not be a substitute for a pilot implementation test in real settings. However, it is possible to use clinical simulations to gain important knowledge concerning work practices, usability and human factors prior to widespread system release, and they can thereby contribute greatly to ensuring patient safety.

## Single-case experiment (N=1 trial)[35–38]

Single-case designs include a family of methods in which each participant serves as his or her own control. In a typical study, some behavior or self-reported symptom is measured repeatedly during all conditions for all participants. The experimenter systematically introduces and withdraws control and intervention conditions and then assesses effects of the intervention on behavior across replications of these conditions within and across participants. Thus, the telltale traits of these studies include repeated and frequent assessment of behavior, experimental manipulation of the independent variable, and replication of effects within and across participants.

## Think aloud method[11]

The think aloud method can be of high value in evaluating a system's design on usability flaws and is therefore frequently used to gather information about a system's usability in testing computer systems with potential end users. During recorded usability sessions, users ‘interact’ with a (prototype) system or interface according to a predetermined set of scenarios while verbalizing their thoughts. Analyses of these verbal reports provide detailed insight into usability problems actually encountered by end users but also in the causes underlying these problems.

## Vignette study[27]

A quantitative vignette study consists of two components: (a) a vignette experiment as the core element, and (b) a traditional survey for the parallel and supplementary measurement of additional respondent-specific characteristics, which are used as covariates in the analysis of vignette data. A vignette is a short, carefully constructed description of a person, object, or situation, representing a systematic combination of characteristics. Within vignette studies, respondents are typically confronted not only with one single vignette but with a whole population of vignettes in order to elicit their beliefs, attitudes, judgments, knowledge, or intended behavior with respect to the presented vignette scenarios. Finally, the aim of a vignette study is to identify and assess the importance of those vignette factors which causally affect individual responses to the contextualized but hypothetical vignette settings.

# Effectiveness (impact)

## Action research[39]

Action research (AR) is used to both understand and assist eHealth implementation in complex social settings. The AR method provides an insightful technique for studying information systems development (ISD) process across time and across technologies and contexts. Defined as “an inquiry into how human beings design and implement action in relation to one another”, the purpose of AR is to observe and create effective organizational change.

## Adaptive design[40,41]*

This is an alternative clinical trial design. The idea is to use accumulating data from the trial to make preplanned changes to the design. Usually, a part of the adaptive design is to specify in advance a predictive model that uses intermediate or surrogate endpoints to predict the final primary effectiveness endpoint, which helps to guide when to stop recruiting more patients unnecessarily into the trial based on posterior predictive probability calculations; this is especially helpful in studies with long-term endpoints when the intermediate endpoints are thought to be predictive.

## Big data analysis[42]*

Overarching term for all kinds of methods used for analysis of ‘big’ datasets. Mostly ‘machine learning’: an umbrella term for techniques that fit models algorithmically by adapting to patterns in data.

## Case series study[28]

Observational study design which describes several patients (cases) over time. Mostly hypothesis forming (early stage of effectiveness research) and without control group or placebo.

## CHEATS: a generic information communication technology (ICT) evaluation[43]

CHEATS is a generic information communication technology (ICT) evaluation framework based on a methodology of formative process evaluation utilizing both quantitative and qualitative methods. CHEATS stand for: Clinical, Human and organizational, Educational, Administrative, Technical, Social.

## Cluster randomized controlled trial[44]*

Randomized controlled trial not randomizing individuals, but ‘cluster’ mostly health care centers, or primary care practices.

## Cohort study (retro- and prospective)[45]*

Observational design, in which groups of patients are followed over time. Usually, multiple exposures and outcomes can be defined in a cohort. Retro-and prospective mostly refers to the timing of data acquisition (before or after designing the study). Patients are sampled on the basis of exposure. Information about baseline characteristics is obtained, and the occurrence of outcomes is assessed during a specified follow-up period. At baseline, all exposed or unexposed persons or both may be included.

## Controlled before-after study / non-randomized controlled trial (CBA / NRCT)[46]*

A study in which observations are made before and after the implementation of an intervention, both in a group that receives the intervention and in a control group that does not.

## Controlled clinical trial (CCT)[47,48]*

A clinical study that includes a comparison (control) group. The comparison group receives a placebo, another treatment, or no treatment at all.

## Cost-effectiveness analysis[49,50]

Cost effectiveness analysis (CEA) produces a numerical ratio—the incremental cost effectiveness ratio—in value (dollars, euro’s) per a gain in health from a measure (for example, years of life (QALY). This ratio is used to express the difference in cost effectiveness between new diagnostic tests or treatments and current ones.

## Cross-sectional study[29]

Observational study design, which samples the exposure and outcome at one moment in time. Useful to get quick insight in possible associations. Drawback is the lack of follow-up time to study relations between exposure and outcome over time.

## Crossover study[51]*

Randomized, parallel group clinical trials often require large groups of patients; this is expensive and takes time. A randomized cross-over trial can be an efficient and more affordable alternative. A cross-over design can be used to study chronic disorders in which treatments have temporary effects. Participants receive all treatments in consecutive periods and outcomes are measured after every period. In general, only a quarter of the total group size is needed for cross-over studies compared with parallel group studies.

## Economic evaluation[52]

Overarching term to describe the methods used for economic evaluation, which include three major categories based on their evaluation method: cost-effectiveness analyses, cost-utility analyses or cost-benefit analyses.

## (Fractional-)factorial (ANOVA) design[53,54]

Evaluation of eHealth treatments often occurs via randomized clinical trials. While there is a vital role for such trials, they often do not provide as much information as alternative experimental strategies. For instance, engineering researchers typically use highly efficient factorial and fractional-factorial designs that allow for the testing of multiple hypotheses or interventions with no loss of power even as the number of tested interventions increases.

## HAS methodological framework[52]

The French national authority for health (HAS) published in 2011, a methodological framework for its economic evaluations. Drawing on its vast experience and the in-depth work on economic evaluation methods within the Economic Evaluation and Public Health Committee, the HAS strives to present and share the principles and methods that it uses in economic evaluation analyses, comparing the health effects to be expected from health care with the resources used to produce such care. In addition to the principles and methods that it uses in economic evaluation analyses, quantitative and qualitative research methods should be combined. This will make it possible to take into account the project’s context and understand the different effects of telemedicine interventions. The technology, the medical field, the application of telemedicine, the objectives and local context will decide important parameters which must be taken into account.

## Interrupted time series analysis[55–58]

Interrupted time series (ITS) analysis is a useful quasi-experimental design with which to evaluate the longitudinal effects of interventions, through regression modelling. The term quasi-experimental refers to an absence of randomization, and ITS analysis is principally a tool for analyzing observational data where full randomization, or a case-control design, is not affordable or possible. Its main advantage over alternative approaches is that it can make full use of the longitudinal nature of the data and account for pre-intervention trends.

## Matched cohort study design[32]

Matching is not uncommon in epidemiological studies and refers to the selection of unexposed subjects’ i.e., controls that in certain important characteristics are identical to cases. Most frequently matching is used in case-control studies but it can also be used in cohort studies. The matching procedure is often directed towards classical background factors such as sex and age.

## Methods comparison study[33]

Two different overarching methodologies for method-comparison studies have been commonly used: equivalence studies and non-inferiority studies. In equivalence studies, we are interested in whether the new assessment does not differ from the conventional (usually in-person) assessment in either direction by a pre-specified amount (i.e. a two-sided test). In an equivalence trial the new assessment method will be selected regardless of whether it is better or worse than an existing assessment as long as the difference falls within the predefined zone of allowable difference (and meets other criteria such as cost effective and stakeholder satisfaction). Commonly in telehealth, the existing model of care (e.g. specialist assessment in tertiary hospital for cognitive impairment) will not be replaced, but rather the telehealth option will be used for people who cannot access conventional services. In this case, the question is whether the telehealth assessment is ‘as good’ as or rather ‘not inferior’ to conventional practice.

## Micro-randomized trial[36,37][59,60]

Micro‐randomized trials are trials in which participants are randomly assigned a treatment from the set of possible treatment actions at several times throughout the day. Thus, each participant may be randomized hundreds or thousands of times over the course of a study. This is very different than a traditional randomized trial, in which participants are randomized once to one of a handful of treatment groups.

## Mixed methods[18,19]*

Mixed methods research (MMR) is an emerging and evolving research methodology that requires both qualitative and quantitative approaches within the same study. It is an approach to research in the social, behavioral and health sciences in which the investigator gathers both quantitative and qualitative data, integrates the two, and then draws interpretations based on the combined strengths of both sets of data to understand research problems. MMR is important for telehealth research because questions that profit most from a mixed methods design tend to be broad, complex and multifaceted.

## Non-inferiority trial[61]*

Demonstrating superiority of the new solution in terms of quality or efficacy of treatment is not always necessary, as the telemedicine/e-health solution/application may have other types of advantages, including saved travel time or saved costs. Testing that the new solution is not inferior to a traditional counterpart may therefore seem to be sufficient in many cases.

## Parallel cohort study with nested RCT[62]

The longitudinal observational cohort study with a nested RCT design has many similarities with the parallel group RCT but embeds the RCT within a cohort study. The main advantage of a nested RCT design is the available follow-up information of those who refuse the intervention or are non-adherent. By having asked informed consent for the observational study before offering the RCT intervention, baseline and follow-up data can be collected from all individuals, including those who refuse the intervention. Furthermore, participants are only eligible for the nested RCT if they have complied with the observational cohort data collection, which ensures that participants randomized are motivated to participate.

## Patient reported outcome measures (PROMs)[63]*

PROMs seek to ascertain patients’ views of their symptoms, their functional status, and their health-related quality of life. PROMs are often wrongly referred to as so called “outcome measures,” though they actually measure health—by comparing a patient’s health at different times, the outcome of the care received can be determined. It’s important to distinguish PROMs from patient reported experience measures (PREMs), which focus on aspects of the humanity of care, such as being treated with dignity or being kept waiting.

## Practical clinical trial (PCT)[64]

There are four key characteristics of practical trials. They study representative patients, are conducted in multiple settings, employ as controls reasonable alternative intervention choices rather than no treatment or “usual care,” and report on outcomes relevant to clinicians, potential adoptees, and policymakers.

## Pragmatic randomized controlled trial (P-RCT)[65,66]*

The term “pragmatic” for RCTs was introduced half a century ago. In contrast to “explanatory” RCTs that test hypotheses on whether the intervention causes an outcome of interest in ideal circumstances, “pragmatic” RCTs aim to provide information on the relative merits of real-world clinical alternatives in routine care. A critical aim of an explanatory RCT is to ensure internal validity (prevention of bias); conversely, a pragmatic RCT focuses on maximizing external validity (generalizability of the results to many real-world settings), but should try to preserve as much internal validity as possible.

## Preference clinical trial (PCT)[34]

In a preference clinical trial (PCT), two or more health-care interventions are compared among several groups of patients, at least some of whom have purposefully chosen the intervention to be administered to them. This stands in contrast to the randomized, controlled clinical trial (RCT), where patients are randomly assigned to receive one of the available test interventions.

## Pretest-posttest study[56,67]*

The basic premise behind the pretest–posttest design involves obtaining a pretest measure of the outcome of interest prior to administering some treatment, followed by a posttest on the same measure after treatment occurs. Pretest–posttest designs are employed in both experimental and quasi-experimental research and can be used with or without control groups. For example, quasi-experimental pretest–posttest designs may or may not include control groups, whereas experimental pretest–posttest designs must include control groups. Furthermore, despite the versatility of the pretest–posttest designs, in general, they still have limitations, including threats to internal validity. Although such threats are of particular concern for quasi-experimental pretest–posttest designs, experimental pretest–posttest designs also contain threats to internal validity.

## Propensity score[40]

The propensity score is the conditional probability of receiving treatment A rather than treatment B, given the observed covariates. Rosenbaum and Rubin (1983) state that the propensity score is a balancing score in the sense that it is a function of the observed covariates such that conditional on the propensity score, the distribution of observed baseline covariates will be similar between the two treatment groups. Then, the propensity score methods can be used to assess treatment group comparability with respect to patient baseline covariates and adjust for imbalances in those covariates to allow for a sensible treatment comparison in clinical outcomes. More importantly, for observational studies in regulatory settings, the methodology can be utilized to design an observational study and mimic RCT in the aspects of study design integrity and interpretability of study results.

## Survey methods[8]*

Surveys are commonly used in telehealth research to assess patient satisfaction, patient experiences, patient preferences and attitudes, and the technical quality of a teleconsultation. The popularity of the survey as a method of measurement can be understood through three major strengths of this technique. First, confidential survey questions are well suited to capture individuals’ experiences, perceptions and attitudes. Second, pre-existing scales can be used across studies, enabling the comparison and replication of results. Third, the validity and reliability of survey instruments can be assessed through rigorous, transparent and well-accepted validation methods, providing the researcher with confidence that the measures tap the intended constructs, and provide an accurate measurement.

## Randomized controlled trial (RCT)[68]

The randomized control trial (RCT) is a trial in which subjects are randomly assigned to one of two groups: one (the experimental group) receiving the intervention that is being tested, and the other (the comparison group or control) receiving an alternative (conventional) treatment. The two groups are then followed up to see if there are any differences between them in outcome. The results and subsequent analysis of the trial are used to assess the effectiveness of the intervention, which is the extent to which a treatment, procedure, or service does patients more good than harm.

## Sequential Multiple Assignment Randomized Trial (SMART)[53,54,65,69,70]

The SMART approach is a randomized experimental design that has been developed especially for building time-varying adaptive interventions. The SMART approach enables the intervention scientist to address questions like these in a holistic yet rigorous manner, taking into account the order in which components are presented rather than considering each component in isolation. A SMART trial provides an empirical basis for selecting appropriate decision rules and tailoring variables. The end goal of the SMART approach is the development of evidence-based adaptive intervention strategies, which are then evaluated in a subsequent RCT.

## Stepped wedge trial[71–73]*

In a stepped wedge design, an intervention is rolled-out sequentially to the trial participants (either as individuals or clusters of individuals) over a number of time periods. The order in which the different individuals or clusters receive the intervention is determined at random and, by the end of the random allocation, all individuals or groups will have received the intervention. Stepped wedge designs incorporate data collection at each point where a new group (step) receives the intervention. Data analysis to determine the overall effectiveness of the intervention subsequently involves comparison of the data points in the control section of the wedge with those in the intervention section. There are two key (non-exclusive) situations in which a stepped wedge design is considered advantageous when compared to a traditional parallel design. First, if there is a prior belief that the intervention will do more good than harm, rather than a prior belief of equipoise, it may be unethical to withhold the intervention from a proportion of the participants, or to withdraw the intervention as would occur in a cross-over design. Second, there may be logistical, practical or financial constraints that mean the intervention can only be implemented in stages.

## Trials of intervention principles (TIPs)[70]*

Trials of Behavioral intervention technologies (BIT) should be viewed as experiments to test principles within that BIT that can then be more broadly applied by developers, designers, and researchers in the creation of BITs and the science behind technology-based behavioral intervention. As such, we refer to these trials as “Trials of Intervention Principles” (TIPs), as they test the theoretical concepts represented within the BIT, rather than the specific technological instantiation of the BIT itself.

## Wait list control group study[74,75]

A wait list control group, also called a wait list comparison, is a group of participants included in an outcome study that is assigned to a waiting list and receives intervention after the active treatment group. This control group serves as an untreated comparison group during the study, but eventually goes on to receive treatment at a later date. Wait list control groups are often used when it would be unethical to deny participants access to treatment, provided the wait is still shorter than that for routine services.

# Implementation (uptake)

## Action research[39]

Action research (AR) is used to both understand and assist eHealth implementation in complex social settings. The AR method provides an insightful technique for studying information systems development (ISD) process across time and across technologies and contexts. Defined as “an inquiry into how human beings design and implement action in relation to one another”, the purpose of AR is to observe and create effective organizational change.

## Big data analysis[42]*

Overarching term for all kinds of methods used for analysis of ‘big’ datasets. Mostly ‘machine learning’: an umbrella term for techniques that fit models algorithmically by adapting to patterns in data.

## CHEATS: a generic information communication technology (ICT) evaluation[43]

CHEATS is a generic information communication technology (ICT) evaluation framework based on a methodology of formative process evaluation utilizing both quantitative and qualitative methods. CHEATS stand for: Clinical, Human and organizational, Educational, Administrative, Technical, Social.

## Economic evaluation[52]

Overarching term to describe the methods used for economic evaluation, which include three major categories based on their evaluation method: cost-effectiveness analyses, cost-utility analyses or cost-benefit analyses.

## Critical incident technique[16]

First described by John C. Flanagan in 1954, the critical incident technique (CIT) is a well-established qualitative research tool used in many areas of the health sciences. Flanagan describes the technique as consisting of “a set of procedures for collecting direct observations of human behavior in such a way as to facilitate their potential usefulness in solving practical problems.” The CIT began its life as an offshoot of the Aviation Psychology Program of the United States Army Air Forces in World War II.

## Focus group[3,4]

A focus group is a group discussion on a particular topic organized for research purposes. This discussion is guided, monitored and recorded by a researcher (sometimes called a moderator or facilitator). Focus groups are used for generating information on collective views, and the meanings that lie behind those views.

## HAS methodological framework[52]

The French national authority for health (HAS) published in 2011, a methodological framework for its economic evaluations. Drawing on its vast experience and the in-depth work on economic evaluation methods within the Economic Evaluation and Public Health Committee, the HAS strives to present and share the principles and methods that it uses in economic evaluation analyses, comparing the health effects to be expected from health care with the resources used to produce such care. In addition to the principles and methods that it uses in economic evaluation analyses, quantitative and qualitative research methods should be combined. This will make it possible to take into account the project’s context and understand the different effects of telemedicine interventions. The technology, the medical field, the application of telemedicine, the objectives and local context will decide important parameters which must be taken into account.

## Interrupted time series analysis[55–58]

Interrupted time series (ITS) analysis is a useful quasi-experimental design with which to evaluate the longitudinal effects of interventions, through regression modelling. The term quasi-experimental refers to an absence of randomization, and ITS analysis is principally a tool for analyzing observational data where full randomization, or a case-control design, is not affordable or possible. Its main advantage over alternative approaches is that it can make full use of the longitudinal nature of the data and account for pre-intervention trends.

## Interview[3]

There are three fundamental types of research interviews: structured, semi-structured and unstructured. Structured interviews are, essentially, verbally administered questionnaires, in which a list of predetermined questions are asked, with little or no variation and with no scope for follow-up questions to responses that warrant further elaboration. Conversely, unstructured interviews do not reflect any preconceived theories or ideas and are performed with little or no organization. Semi-structured interviews consist of several key questions that help to define the areas to be explored, but also allows the interviewer or interviewee to diverge in order to pursue an idea or response in more detail.

## Logfile analysis[76,77]

Transaction log data provides ‘real-time’ use statistics that document the specific steps in individuals’ information searches and thus direct evidence of interactions between user and online eHealth resources. logs provide fairly abundant evidence of specific resource use and with log analysis researchers can collect information about the actual use of a system (e.g., every keystroke and machine response) in such an unobtrusive way. This enables researchers to better understand the way in which users interact with computers and content.

## Methods comparison study[33]

Two different overarching methodologies for method-comparison studies have been commonly used: equivalence studies and non-inferiority studies. In equivalence studies, we are interested in whether the new assessment does not differ from the conventional (usually in-person) assessment in either direction by a pre-specified amount (i.e. a two-sided test). In an equivalence trial the new assessment method will be selected regardless of whether it is better or worse than an existing assessment as long as the difference falls within the predefined zone of allowable difference (and meets other criteria such as cost effective and stakeholder satisfaction). Commonly in telehealth, the existing model of care (e.g. specialist assessment in tertiary hospital for cognitive impairment) will not be replaced, but rather the telehealth option will be used for people who cannot access conventional services. In this case, the question is whether the telehealth assessment is ‘as good’ as or rather ‘not inferior’ to conventional practice.

## Mixed methods[18,19]*

Mixed methods research (MMR) is an emerging and evolving research methodology that requires both qualitative and quantitative approaches within the same study. It is an approach to research in the social, behavioral and health sciences in which the investigator gathers both quantitative and qualitative data, integrates the two, and then draws interpretations based on the combined strengths of both sets of data to understand research problems. MMR is important for telehealth research because questions that profit most from a mixed methods design tend to be broad, complex and multifaceted.

## Normalization process model[78]

Normalization is defined as the embedding of a technique, technology or organizational change as a routine and taken-for-granted element of clinical practice. The normalization process model offers a means of conceptualizing complex interventions in practice. It focuses on interactions within and between processes of practice, (characterized as endogenous and exogenous) and is thus not intended to compete with wider conceptual models of innovation diffusion or of network behavior in organizations. The model takes as its starting point the points of contact between four domains: (i) the interactional work that professionals and patients do within the clinical encounter and its temporal order, (interactional workability); (ii) the embeddedness of trust in professional knowledge and practice, (relational integration); (iii) the organizational distribution of work, knowledge and practice across divisions of labor (skill set workability); and, (iv) its contexts of institutional location and organizational capacity, (contextual integration).

## Patient reported outcome measures (PROMs)[63]*

PROMs seek to ascertain patients’ views of their symptoms, their functional status, and their health-related quality of life. PROMs are often wrongly referred to as so called “outcome measures,” though they actually measure health—by comparing a patient’s health at different times, the outcome of the care received can be determined. It’s important to distinguish PROMs from patient reported experience measures (PREMs), which focus on aspects of the humanity of care, such as being treated with dignity or being kept waiting.

## Survey methods[8]*

Surveys are commonly used in telehealth research to assess patient satisfaction, patient experiences, patient preferences and attitudes, and the technical quality of a teleconsultation. The popularity of the survey as a method of measurement can be understood through three major strengths of this technique. First, confidential survey questions are well suited to capture individuals’ experiences, perceptions and attitudes. Second, pre-existing scales can be used across studies, enabling the comparison and replication of results. Third, the validity and reliability of survey instruments can be assessed through rigorous, transparent and well-accepted validation methods, providing the researcher with confidence that the measures tap the intended constructs, and provide an accurate measurement.

## Sociotechnical evaluation[79]

Sociotechnical perspectives assume that ‘organizational and human (socio) factors and information technology system factors (technical) are inter-related parts of one system, each shaping the other’. In line with this, sociotechnical evaluations involve researching the way technical and social dimensions change and shape each other over time. A further defining component of sociotechnical evaluations is the attempt to study processes associated with the introduction of a new technology in social/organizational settings, as these mediators can offer important insights into potentially transferable lessons. This focus on processes is important, because of the increasing number of technological functionalities and vast differences in implementation contexts. In contrast, evaluations that focus solely on investigating the impact of technology on outcomes often have limited generalizability beyond the immediate clinical setting in which the research was undertaken.

# All phases methods

## CeHRes Roadmap[7,80]

This roadmap serves as a practical guideline to help plan, coordinate, and execute the participatory development process of eHealth technologies. The framework is meant for developers, researchers, and policy makers and for educational purposes. It also serves as an analytical instrument for decision making about the use of eHealth technologies.

## Continuous evaluation of evolving behavioral intervention technologies (CEEBIT)[38,81]

A methodologic framework that can support the evaluation of multiple Behavioral intervention technologies (BITs) or evolving versions, eliminating those that demonstrate poorer outcomes, while allowing new BITs to be entered at any time. CEEBIT could be used to ensure the effectiveness of BITs provided through deployment platforms in clinical care organizations.

## Five-stage model for comprehensive research on telehealth[82]

A five-stage model as a framework for planning a comprehensive telehealth research program for a new intervention or service system. The stages are: (1) Concept development, (2) Service design, (3) Pre-implementation, (4) Implementation, (5) Post-implementation.

## Life-cycle–based approach[83]

The overall aim of this model is to maximize the benefits while minimizing any risks associated with the eHealth intervention. This balance is achieved by iterative formative evaluations at four key stages of the eHealth intervention's lifecycle: I inception, II requirements and analysis, III design develop and test, IV implement and deploy. This model has the additional advantage of providing a means to understand the implementation process.

## mHealth Agile and User-Centered Research and Development Lifecycle[84]

This mHealth research model mirrors traditional clinical research methods in its attention to safety and efficacy, while also accommodating the rapid and iterative development and evaluation required to produce effective, evidence-based, and sustainable digital products. It consists of a project identification stage followed by four phases of clinical evaluation: Phase 1: User Experience Design, Development, & Alpha Testing; Phase 2: Beta testing; Phase 3: Clinical Trial Evaluation; and Phase 4: Post-Market Surveillance. These phases include sample gating questions and are adapted to accommodate the unique nature of digital product development.

## mHealth Development and Evaluation Framework[85]

The mHealth Framework includes six stages, some of which may be implemented concurrently: first, conceptualization, to determine the theoretical basis and empirical foundation of a new intervention; second, formative research, to gauge target audience response and refine the concept; third, pre-testing, to determine the intervention’s acceptability, and further refine the intervention; fourth, pilot testing, involving a small non-randomized study to test feasibility of the intervention and study processes (e.g., recruitment and data collection); fifth, randomized controlled trial, to test the effect of the intervention in comparison with a control group(s); and sixth, qualitative research, for further refinement before moving to a more scaled-up intervention.

## Model for Assessment of Telemedicine applications (MAST)[86,87]

The Model for Assessment of Telemedicine (MAST) focuses on the measurement of effectiveness and quality of care. MAST represents a multidisciplinary process, evaluating the medical, social, economic, and ethical aspects of telemedicine in a systematic, unbiased, robust manner. The use of MAST includes three steps: preceding assessment (Step 1) the maturity of the telemedicine technology and the organization using the service is assessed before the assessment of effectiveness is carried out; multidisciplinary assessment (Step 2) of the effectiveness of the technology by encompassing seven domains, and an assessment should be made of the transferability of the results (Step 3).

## Multiphase Optimization Strategy (MOST)[54,85]

MOST is an alternative way of building, optimizing, and evaluating eHealth interventions. It incorporates the standard RCT, but before the RCT is undertaken MOST also uses a principled method for identifying which components are active in an intervention and which levels of each component lead to the best outcomes. The principles underlying MOST are drawn from engineering, and emphasize efficiency. The MOST method consists of three phases, each of which addresses a different set of questions about the intervention by means of randomized experimentation.

## Proposed Framework for Evaluating mHealth Services[88]

The proposed framework, includes three main stages named as Service Requirement Analysis, Service Development and Service Delivery. The iterative nature of the proposed framework guarantees continuous improvement of m-health services. Moreover, important evaluation dimensions including technical, organizational, social and legal, strategic and usability as well as effects of key stakeholders of m-Health service on mentioned dimensions have been considered in the proposed framework.

## RE-AIM framework (Reach, Effectiveness, Adoption, Implementation and Maintenance)[64,89,90]*

The RE-AIM model has been widely used to plan, evaluate and review health promotion and disease management interventions. RE-AIM is a conceptual model designed to enhance the quality, speed, and public health impact of efforts to move from research into long-term effectiveness in real-world settings. It may be particularly useful for increasing the potential of eHealth interventions intended to be translated into practice. RE-AIM consists of five evaluative dimensions related to both internal and external validity: Reach, Efficacy/Effectiveness, Adoption, Implementation, and Maintenance and is intended for use at all stages of research, from planning to evaluation.

## Stage Model of Behavioral Therapies Research[65,91]

The Stage Model of Behavioral Therapies Research articulates three progressive stages of development and evaluation of behavioral interventions. This model is especially relevant to Web-based intervention research given its goals of encouraging innovation and facilitating widespread use of empirically validated behavioral programs. Stage I consists of pilot/feasibility testing, manual writing, training program development, and adherence/competence measure development for new and untested treatments. Stage II initially consists of randomized clinical trials (RCTs) to evaluate efficacy of manualized and pilot-tested treatments which have shown promise or efficacy in earlier studies. Stage III consists of studies to evaluate transportability of treatments for which efficacy has been demonstrated in at least two RCTs. Key stage III research issues revolve around generalizability; implementation; cost effectiveness issues; and consumer/marketing issues.

## Stead’s et al. evaluation framework[92,93]

The premises of the Stead et al. (1994) framework are that evaluation is essential to each of the five stages of system development and that the level of evaluation should be well matched to the development stage. The appropriate type of evaluation will vary according to the stage of work, but all evaluations must be rigorous and systematic. The stages of development correspond to a standard software design life cycle that begins with system specification and concludes with routine use of a product. The levels of evaluation present a range of methods to apply at each stage of development. For example, formative methods (e.g., needs requirement) are used in the earlier stages, and a more summative approach to evaluate the validity and efficacy of a system (e.g., a controlled clinical trial) is used in the later stages.

# References

1. Van Engen-Verheul M, Peek N, Vromen T, Jaspers M, De Keizer N. How to use concept mapping to identify barriers and facilitators of an electronic quality improvement intervention. Stud Health Technol Inform 2015. PMID:25991112

2. ENAQ and EQET [Internet]. [cited 2019 Oct 1]. Available from: http://healthliteracycentre.eu/key-success-factors-for-e-health-apps/

3. Gill P, Stewart K, Treasure E, Chadwick B. Methods of data collection in qualitative research: Interviews and focus groups. Br Dent J 2008; PMID:18356873

4. Peeters JM, Krijgsman JW, Brabers AE, Jong JD De, Friele RD. Use and Uptake of eHealth in General Practice: A Cross-Sectional Survey and Focus Group Study Among Health Care Users and General Practitioners. JMIR Med Informatics 2016; PMID:27052805

5. Swinkels ICS, Huygens MWJ, Schoenmakers TM, Nijeweme-D’Hollosy WO, Velsen L van, Vermeulen J, Schoone-Harmsen M, Jansen YJFM, Van Schayck OCP, Friele R, De Witte L. Lessons learned from a living lab on the broad adoption of eHealth in primary health care. J Med Internet Res. 2018. PMID: 29599108

6. Kramer-Jackman KL, Popkess-Vawter S. Method for technology-delivered healthcare measures. CIN - Comput Informatics Nurs 2011; PMID:10066602

7. Alpay L, Doms R, Bijwaard H. Embedding persuasive design for self-health management systems in Dutch healthcare informatics education: Application of a theory-based method. Health Informatics J 2018; PMID:30192696

8. Langbecker D, Caffery LJ, Gillespie N, Smith AC. Using survey methods in telehealth research: A practical guide. J Telemed Telecare 2017/07/22. 2017;23(9):770–779. PMID:28728502

9. Harker J, Kleijnen J. What is a rapid review? A methodological exploration of rapid reviews in Health Technology Assessments. Int J Evid Based Healthc 2012;10(4):397–410. PMID:23173665

10. Systematic review - cochrane definition [Internet]. [cited 2019 Oct 1]. Available from: https://consumers.cochrane.org/what-systematic-review

11. Jaspers MWM. A comparison of usability methods for testing interactive health technologies: Methodological aspects and empirical evidence. Int J Med Inform 2009; PMID:19046928

12. A/B testing - definition [Internet]. [cited 2019 Oct 1]. Available from: https://www.optimizely.com/optimization-glossary/ab-testing/

13. Gabarron E, Serrano JA, Fernandez-Luque L, Wynn R, Schopf T. Randomized trial of a novel game-based appointment system for a university hospital venereology unit: Study protocol. BMC Med Inform Decis Mak 2015; [doi: 10.1186/s12911-015-0143-9]

14. Kushniruk AW, Patel VL. Cognitive and usability engineering methods for the evaluation of clinical information systems. J Biomed Inform. 2004. PMID:15016386

15. Khajouei R, Zahiri Esfahani M, Jahani Y. Comparison of heuristic and cognitive walkthrough usability evaluation methods for evaluating health information systems. J Am Med Inf Assoc 2016/08/09. 2017;24(e1):e55–e60. PMID:27497799

16. FitzGerald K, Seale NS, Kerins CA, McElvaney R. The critical incident technique: A useful tool for conducting qualitative research. J Dent Educ. 2008. PMID:18316534

17. Henkemans OAB, Dusseldorp EML, Keijsers JFEM, Kessens JM, Neerincx MA, Otten W. Validity and reliability of the eHealth analysis and steering instrument. J Med Internet Res 2013; PMID:25075243

18. Caffery LJ, Martin-Khan M, Wade V. Mixed methods for telehealth research. J Telemed Telecare 2017; PMID:27591744

19. Lee S, Smith CAM. Criteria for quantitative and qualitative data integration: Mixed-methods research methodology. CIN - Comput Informatics Nurs 2012; [doi: 10.1097/NXN.0b013e31824b1f96]

20. Borycki E, Dexheimer JW, Hullin Lucay Cossio C, Gong Y, Jensen S, Kaipio J, Kennebeck S, Kirkendall E, Kushniruk AW, Kuziemsky C, Marcilly R, Rohrig R, Saranto K, Senathirajah Y, Weber J, Takeda H. Methods for Addressing Technology-induced Errors: The Current State. Yearb Med Inf 2016/11/11. 2016;(1):30–40. PMID:27830228

21. Clemensen J, Rothmann MJ, Smith AC, Caffery LJ, Danbjorg DB. Participatory design methods in telemedicine research. J Telemed Telecare 2016/12/29. 2017;23(9):780–785. PMID:28027678

22. Ammenwerth E, Hackl WO, Binzer K, Christoffersen TEH, Jensen S, Lawton K, Skjoet P, Nohr C. Simulation studies for the evaluation of health information technologies: Experiences and results. Heal Inf Manag J 2012; PMID:28683687

23. Jensen S, Kushniruk AW, Nøhr C. Clinical simulation: A method for development and evaluation of clinical information systems. J Biomed Inform 2015; [doi: 10.1016/j.jbi.2015.02.002]

24. Rönnby S, Lundberg O, Fagher K, Jacobsson J, Tillander B, Gauffin H, Hansson PO, Dahlström Ö, Timpka T. mHealth self-report monitoring in competitive middle-and long-distance runners: Qualitative study of long-term use intentions using the technology acceptance model. JMIR mHealth uHealth 2018; PMID: 30104183

25. Portz JD, Bayliss EA, Bull S, Boxer RS, Bekelman DB, Gleason K, Czaja S. Using the technology acceptance model to explore user experience, intent to use, and use behavior of a patient portal among older adults with multiple chronic conditions: Descriptive qualitative study. J Med Internet Res 2019; PMID:30958272

26. Bastien JMC. Usability testing: a review of some methodological and technical aspects of the method. Int J Med Inform 2010; PMID:19345139

27. Atzmüller C, Steiner PM. Experimental vignette studies n survey research. Methodology 2010; [doi: 10.1027/1614-2241/a000014]

28. Holubova A, Vlasakova M, Muzik J, Broz J. Customizing the types of technologies used by patients with type 1 diabetes mellitus for diabetes treatment: Case series on patient experience. J Med Internet Res 2019; PMID:31290400

29. Hansen AH, Broz J, Claudi T, Årsand E. Relations Between the Use of Electronic Health and the Use of General Practitioner and Somatic Specialist Visits in Patients With Type 1 Diabetes: Cross-Sectional Study. J Med Internet Res 2018; PMID:30404766

30. Arain M, Campbell MJ, Cooper CL, Lancaster GA. What is a pilot or feasibility study? A review of current practice and editorial policy. BMC Med Res Methodol 2010; [doi: 10.1186/1471-2288-10-67]

31. Seto E, Morita PP, Tomkun J, Lee TM, Ross H, Reid-Hughian C, Kaboff A, Mulholland D, Cafazzo JA. Implementation of a heart failure telemonitoring system in home care nursing: Feasibility study. J Med Internet Res 2019; PMID:31350841

32. Faresjö T, Faresjö Å. To match or not to match in epidemiological studies - Same outcome but less power. Int J Environ Res Public Health 2010; PMID:20195449

33. Russell TG, Martin-Khan M, Khan A, Wade V. Method-comparison studies in telehealth: Study design and analysis considerations. J Telemed Telecare 2017; PMID:28893117

34. Kowalski CJ, Joel Mrdjenovich A. Patient preference clinical trials: Why and when they will sometimes be preferred. Perspect Biol Med 2013; [doi: 10.1353/pbm.2013.0004]

35. Dallery J, Cassidy RN, Raiff BR. Single-case experimental designs to evaluate novel technology-based health interventions. J Med Internet Res 2013; PMID: 23399668

36. Dempsey W, Liao P, Klasnja P, Nahum-Shani I, Murphy SA. Randomised trials for the Fitbit generation. Significance 2015; [doi: 10.1111/j.1740-9713.2015.00863.x]

37. Klasnja P, Hekler EB, Shiffman S, Boruvka A, Almirall D, Tewari A, Murphy SA. Microrandomized trials: An experimental design for developing just-in-time adaptive interventions. Heal Psychol 2015; PMID:26651463

38. Nicholas J, Boydell K, Christensen H. mHealth in psychiatry: Time for methodological change. Evid Based Ment Health 2016; PMID:27044849

39. Chiasson M, Reddy M, Kaplan B, Davidson E. Expanding multi-disciplinary approaches to healthcare information technologies: What does information systems offer medical informatics? Int J Med Inform 2007; PMID:16769245

40. Campbell G, Yue LQ. Statistical innovations in the medical device world sparked by the FDA. J Biopharm Stat 2016; [doi: 10.1080/10543406.2015.1092037]

41. Law LM, Wason JMS. Design of telehealth trials - Introducing adaptive approaches. Int J Med Inform. 2014. [doi: 10.1016/j.ijmedinf.2014.09.002]

42. Mooney SJ, Pejaver V. Big Data in Public Health: Terminology, Machine Learning, and Privacy. Annu Rev Public Health 2018; [doi: 10.1146/annurev-publhealth-040617-014208]

43. Shaw NT. “CHEATS”: A generic information communication technology (ICT) evaluation framework. Comput Biol Med 2002; [doi: 10.1016/S0010-4825(02)00016-1]

44. Oliveira-Ciabati L, Vieira CS, Franzon ACA, Alves D, Zaratini FS, Braga GC, Sanchez JAC, Bonifacio LP, Andrade MS, Fernandes M, Quintana SM, Fabio S V, Pileggi VN, Vieira EM, Souza JP. PRENACEL - a mHealth messaging system to complement antenatal care: a cluster randomized trial. Reprod Heal 2017/11/09. 2017;14(1):146. PMID:29116028

45. Vandenbroucke JP. Prospective or retrospective: What’s in a name? Br Med J. 1991. [doi: 10.1136/bmj.302.6775.528-d]

46. Controlled before-after study - definition [Internet]. [cited 2019 Oct 1]. Available from: https://childhoodcancer.cochrane.org/non-randomised-controlled-study-nrs-designs

47. van der Meij E, Anema JR, Leclercq WKG, Bongers MY, Consten ECJ, Schraffordt Koops SE, van de Ven PM, Terwee CB, van Dongen JM, Schaafsma FG, Meijerink WJHJ, Bonjer HJ, Huirne JAF. Personalised perioperative care by e-health after intermediate-grade abdominal surgery: a multicentre, single-blind, randomised, placebo-controlled trial. Lancet 2018; [doi: 10.1016/S0140-6736(18)31113-9]

48. Controlled clinical trial - definition [Internet]. [cited 2019 Oct 1]. Available from: https://www.cancer.gov/publications/dictionaries/cancer-terms/def/controlled-clinical-trial

49. de la Torre-Díez I, López-Coronado M, Vaca C, Aguado JS, de Castro C. Cost-Utility and Cost-Effectiveness Studies of Telemedicine, Electronic, and Mobile Health Systems in the Literature: A Systematic Review. Telemed e-Health 2015; PMID:25474190

50. Elbert NJ, Van Os-Medendorp H, Van Renselaar W, Ekeland AG, Hakkaart-Van Roijen L, Raat H, Nijsten TEC, Pasmans SGMA. Effectiveness and cost-effectiveness of ehealth interventions in somatic diseases: A systematic review of systematic reviews and meta-analyses. J Med Internet Res. 2014. PMID: 24739471

51. Bonten TN, Siegerink B, Van Der Bom JG. Cross-overstudies. Ned Tijdschr Geneeskd 2013; PMID:23328025

52. Bongiovanni-Delaroziere I, Le Goff-Pronost M. Economic evaluation methods applied to telemedicine: From a literature review to a standardized framework. Eur Res Telemed [Internet] 2017;6(3–4):117–135. PMID:618556690

53. Baker TB, Gustafson DH, Shah D. How can research keep up with eHealth? Ten strategies for increasing the timeliness and usefulness of Ehealth research. J Med Internet Res. 2014. PMID:24554442

54. Collins LM, Murphy SA, Strecher V. The Multiphase Optimization Strategy (MOST) and the Sequential Multiple Assignment Randomized Trial (SMART). New Methods for More Potent eHealth Interventions. Am J Prev Med 2007; PMID:17466815

55. Chumbler NR, Kobb R, Brennan DM, Rabinowitz T. Recommendations for Research Design of Telehealth Studies. Telemed e-Health 2008; PMID:19035813

56. Grigsby J, Bennett RE. Alternatives to randomized controlled trials in telemedicine. J Telemed Telecare 2006; [doi: 10.1258/135763306778393162]

57. Liu JLY, Wyatt JC. The case for randomized controlled trials to assess the impact of clinical information systems. J Am Med Informatics Assoc 2011; PMID:21270132

58. Kontopantelis E, Doran T, Springate DA, Buchan I, Reeves D. Regression based quasi-experimental approach when randomisation is not an option: Interrupted time series analysis. BMJ 2015; PMID:26058820

59. Law LM, Edirisinghe N, Wason JMS. Use of an embedded, micro-randomised trial to investigate non-compliance in telehealth interventions. Clin Trials 2016; [doi: 10.1177/1740774516637075]

60. Walton A, Nahum-Shani I, Crosby L, Klasnja P, Murphy S. Optimizing Digital Integrated Care via Micro-Randomized Trials. Clin Pharmacol Ther [Internet] 2018;104(1):53–58. PMID:622580572

61. Kummervold PE, Johnsen JAK, Skrøvseth SO, Wynn R. Using noninferiority tests to evaluate telemedicine and e-health services: Systematic review. J Med Internet Res. 2012. PMID:23022989

62. Younge JO, Kouwenhoven-Pasmooij TA, Freak-Poli R, Roos-Hesselink JW, Hunink MGM. Randomized study designs for lifestyle interventions: A tutorial. Int J Epidemiol 2015; [doi: 10.1093/ije/dyv183]

63. Black N. Patient reported outcome measures could help transform healthcare. BMJ 2013; [doi: 10.1136/bmj.f167]

64. Glasgow RE. eHealth Evaluation and Dissemination Research. Am J Prev Med 2007; [doi: 10.1016/j.amepre.2007.01.023]

65. Danaher BG, Seeley JR. Methodological issues in research on web-based behavioral interventions. Ann Behav Med. 2009. PMID:19806416

66. Dal-Ré R, Janiaud P, Ioannidis JPA. Real-world evidence: How pragmatic are randomized controlled trials labeled as pragmatic? BMC Med 2018; [doi: 10.1186/s12916-018-1038-2]

67. Salkind NJ. Encyclopedia of Research Design: Pretest–Posttest Design. Encycl Res Des 2010; [doi: https://dx.doi.org/10.4135/9781506326139.n538]

68. Kendall JM. Designing a research project: Randomised controlled trials and their principles. Emerg Med J. 2003.

69. Almirall D, Nahum-Shani I, Sherwood NE, Murphy SA. Introduction to SMART designs for the development of adaptive interventions: with application to weight loss research. Transl Behav Med 2014; PMID:25264466

70. Mohr DC, Schueller SM, Riley WT, Brown CH, Cuijpers P, Duan N, Kwasny MJ, Stiles-Shields C, Cheung K. Trials of intervention principles: Evaluation methods for evolving behavioral intervention technologies. J Med Internet Res 2015; PMID:26155878

71. Brown CA, Lilford RJ. The stepped wedge trial design: A systematic review. BMC Med Res Methodol. 2006. PMID:17092344

72. Hussey MA, Hughes JP. Design and analysis of stepped wedge cluster randomized trials. Contemp Clin Trials 2007; PMID:16829207

73. Spiegelman D. Evaluating public health interventions: 2. Stepping up to routine public health evaluation with the stepped wedge design. Am J Public Health 2016; PMID:26885961

74. Nguyen HQ, Cuenco DA, Wolpin S, Benditt J, Carrieri-Kohlman V. Methodological considerations in evaluating eHealth interventions. Can J Nurs Res. 2007. PMID:17450708

75. Elliott SA, Brown JSL. What are we doing to waiting list controls? Behav Res Ther 2002; [doi: 10.1016/S0005-7967(01)00082-1]

76. Han JY. Transaction logfile analysis in health communication research: Challenges and opportunities. Patient Educ Couns 2011; PMID:21277146

77. Sieverink F, Kelders S, Poel M, van Gemert-Pijnen L. Opening the Black Box of Electronic Health: Collecting, Analyzing, and Interpreting Log Data. JMIR Res Protoc 2017; PMID:28784592

78. May C. A rational model for assessing and evaluating complex interventions in health care. BMC Health Serv Res 2006; PMID:16827928

79. Cresswell KM, Sheikh A. Undertaking sociotechnical evaluations of health information technologies. Inf Prim Care. 2014/05/21. 2014;21(2):78–83. PMID:24841408

80. van Gemert-Pijnen JEWC, Nijland N, van Limburg M, Ossebaard HC, Kelders SM, Eysenbach G, Seydel ER. A holistic framework to improve the uptake and impact of eHealth technologies. J Med Internet Res. 2011. PMID:22155738

81. Mohr DC, Cheung K, Schueller SM, Brown CH, Duan N. Continuous evaluation of evolving behavioral intervention technologies. Am J Prev Med 2013; PMID:24050429

82. Fatehi F, Smith AC, Maeder A, Wade V, Gray LC. How to formulate research questions and design studies for telehealth assessment and evaluation. J Telemed Telecare 2017/10/27. 2017;23(9):759–763. PMID:29070001

83. Catwell L, Sheikh A. Evaluating eHealth Interventions: The Need for Continuous Systemic Evaluation. PLoS Med 2009; PMID:19688038

84. Wilson K, Bell C, Wilson L, Witteman H. Agile research to complement agile development: a proposal for an mHealth research lifecycle. npj Digit Med [Internet] 2018;1 (1) (no(46). PMID:624171413

85. Jacobs MA, Graham AL. Iterative development and evaluation methods of mHealth behavior change interventions. Curr Opin Psychol [Internet] 2016;9:33–37. [doi: http://dx.doi.org/10.1016/j.copsyc.2015.09.001]

86. Kidholm K, Clemensen J, Caffery LJ, Smith AC. The Model for Assessment of Telemedicine (MAST): A scoping review of empirical studies. J Telemed Telecare 2017/08/02. 2017;23(9):803–813. PMID:28758525

87. Kidholm K, Jensen LK, Kjolhede T, Nielsen E, Horup MB. Validity of the Model for Assessment of Telemedicine: A Delphi study. J Telemed Telecare [Internet] 2018;24(2):118–125. PMID:623648434

88. Sadegh SS, Khakshour Saadat P, Sepehri MM, Assadi V. A framework for m-health service development and success evaluation. Int J Med Inf [Internet] 2018;112:123–130. PMID:620539989

89. Glasgow RE, Phillips SM, Sanchez MA. Implementation science approaches for integrating eHealth research into practice and policy. Int J Med Inform 2014; [doi: 10.1016/j.ijmedinf.2013.07.002]

90. Glasgow R, Vogt T, Boles S. Evaluating the public health impact of health promotion interventions: the RE-AIM framework. Am J Public Health [Internet] 1999;89(9):1322–1327. PMID:10474547

91. Onken LS, Blaine JD, Battjes RJ. Behavioral therapy research: A conceptualization of a process. In S. W. Henggeler, & A. B. Santos (Eds.), Innovative approaches for difficult-to-treat populations (pp. 477 – 485). Washington, DC: American Psychiatric Press

92. Kaufman D, Roberts WD, Bakken S, Lai T-Y, Merrill J. Applying an Evaluation Framework for Health Information System Design, Development, and Implementation. Nurs Res 2006; [doi: 10.1097/00006199-200603001-00007]

93. Stead WW, Haynes RB, Fuller S, Friedman CP, Travis LE, Beck JR, Fenichel CH, Chandrasekaran B, Buchanan BG, Abola EE, Sievert MC, Gardner RM, Messerle J, Jaffe CC, Pearson WR, Abarbanel RM. Designing medical informatics research and library-resource projects to increase what is learned. J Am Med Informatics Assoc 1994; [doi: 10.1136/jamia.1994.95236134]

# Appendix

| **Evaluation approach** | **Literature**  **map** | **Concept mapping** |
| --- | --- | --- |
| Action research | x | x |
| A/B testing |  | x |
| Adaptive design* | x |  |
| Big data analysis* |  | x |
| Case series study |  | x |
| CeHRes Roadmap | x |  |
| CHEATS: a generic information communication technology (ICT) evaluation | x |  |
| Cluster randomized controlled trial* |  | x |
| Cognitive task analysis (CTA) | x |  |
| Cognitive walkthrough | x | x |
| Cohort study (retro- and prospective)* |  | x |
| Concept mapping study | x |  |
| Continuous evaluation of evolving behavioral intervention technologies (CEEBIT) | x | x |
| Controlled clinical trial (CCT)* |  | x |
| Controlled before-after study (CBA) / non-randomized controlled trial (NRCT)* |  | x |
| Cost-effectiveness analysis |  | x |
| Critical incident technique |  | x |
| Crossover study* |  | x |
| Cross-sectional study |  | x |
| Economic evaluation | x |  |
| eHealth Analysis and Steering Instrument (eASI) |  | x |
| eHealth Needs Assessment Questionnaire (ENAQ) |  | x |
| Evaluative Questionnaire for E-health Tools (EQET) |  | x |
| Feasibility study* |  | X |
| Five-stage model for comprehensive research on telehealth | x |  |
| Focus group |  | x |
| (Fractional-)factorial (ANOVA) design | x | x |
| HAS methodological framework | x |  |
| Heuristic evaluation | x | x |
| Interrupted time series analysis | x | x |
| Interview |  | x |
| Life-cycle–based approach | x |  |
| Living lab |  | x |
| Logfile analysis | x | x |
| Matched cohort study | x |  |
| Method for technology-delivered Healthcare Measures | x |  |
| Methods comparison study |  | x |
| mHealth Agile and User-Centered Research and Development Lifecycle | x |  |
| mHealth Development and Evaluation Framework | x |  |
| Micro-randomized trial | x | x |
| Mixed methods* | x | x |
| Model for Assessment of Telemedicine applications (MAST) | x | x |
| Model of Fogg | x |  |
| Model of Oinas-Kukkonen | x |  |
| Multiphase Optimization Strategy (MOST) | x | x |
| Non-inferiority trial* | x |  |
| Normalization process model | x | x |
| Parallel cohort study with nested RCT |  | x |
| Participatory study | x |  |
| Patient reported outcome measures (PROMs)* |  | x |
| Practical clinical trial (PCT) | x |  |
| Pragmatic randomized controlled trial (P-RCT)* | x | x |
| Preference clinical trial (PCT) |  | x |
| Pretest-posttest study* | x | x |
| Propensity score | x |  |
| Proposed Framework for Evaluating mHealth Services | x |  |
| Randomized controlled trial* |  | x |
| Rapid Review | x | x |
| RE-AIM framework* | x | x |
| Sequential Multiple Assignment Randomized Trial (SMART) | x | x |
| Simulation study | x |  |
| Single-case experiment (N=1 trial) | x | x |
| Sociotechnical evaluation | x |  |
| Stage Model of Behavioral Therapies Research | x |  |
| Stead’s et al. evaluation framework | x |  |
| Stepped wedge trial* | x | x |
| Survey methods* | x | x |
| Systematic review* |  | X |
| Technology Acceptance Model (TAM) | x |  |
| Think aloud method | x | x |
| Trials of intervention principles (TIPs)* | x | x |
| User-centered design (UDC) methods | x | x |
| User-based evaluation | x |  |
| Vignette study |  | x |
| Wait list control group study | x |  |
